# RNA N6-Methyladenosine Responds to Low-Temperature Stress in Tomato Anthers

**DOI:** 10.3389/fpls.2021.687826

**Published:** 2021-06-04

**Authors:** Dandan Yang, Huachao Xu, Yue Liu, Mengzhuo Li, Muhammad Ali, Xiangyang Xu, Gang Lu

**Affiliations:** ^1^Department of Horticulture, Zhejiang University, Hangzhou, China; ^2^College of Horticulture, Northeast Agricultural University, Harbin, China; ^3^Key Laboratory of Horticultural Plant Growth, Development and Quality Improvement, Ministry of Agricultural, Zhejiang University, Hangzhou, China

**Keywords:** abscisic acid, m^6^A, moderate low temperature, pollen development, tomato anther

## Abstract

Cold stress is a serious threat to subtropical crop pollen development and induces yield decline. N6-methyladenosine (m^6^A) is the most frequent mRNA modification and plays multiple physiological functions in plant development. However, whether m^6^A regulates pollen development is unclear, and its putative role in cold stress response remains unknown. Here, we observed that moderate low-temperature (MLT) stress induced pollen abortion in tomato. This phenotype was caused by disruption of tapetum development and pollen exine formation, accompanied by reduced m^6^A levels in tomato anther. Analysis of m^6^A-seq data revealed 1,805 transcripts displayed reduced m^6^A levels and 978 transcripts showed elevated m^6^A levels in MLT-stressed anthers compared with those in anthers under normal temperature. These differentially m^6^A enriched transcripts under MLT stress were mainly related to lipid metabolism, adenosine triphosphatase (ATPase) activity, and ATP-binding pathways. An ATP-binding transcript, *SlABCG31*, had significantly upregulated m^6^A modification levels, which was inversely correlated to the dramatically downregulated expression level. These changes correlated with higher abscisic acid (ABA) levels in anthers and disrupted pollen wall formation under low-temperature stress. Our findings characterized m^6^A as a novel layer of complexity in gene expression regulation and established a molecular link between m^6^A methylation and tomato anther development under low-temperature conditions.

## Introduction

In flowering plants, male reproductive development is vulnerable to abiotic stress (Al Mamun et al., 2010; De Storme and Geelen, 2014; Sharma and Nayyar, 2016; Kiran et al., 2019). Frequent temperature fluctuations cause male sterility in most crops, leading to lower fruit and seed set and final yield (Pacini and Dolferus, 2019). The male gametogenesis of plants depends on a series of complicated processes that lead to the development of premeiotic pollen mother cells into mature pollen grains. The male gametogenesis stage most sensitive to temperature stress is the transition from tetrad to uninucleate microspore (Kim et al., 2001; Oliver et al., 2005; Oda et al., 2010).

Increasing evidence indicates that male sterility induced by low- or high-temperature stresses is linked to tapetal dysfunction. The tapetum is the innermost sporophytic cell layer of the anther wall; it provides nutrients and precursors for microsporogenesis and pollen wall formation through programmed cell death (PCD)-triggered degradation (Ma, 2005; Ariizumi and Toriyama, 2011). The tapetum is the most susceptible to temperature fluctuations at the young microspore stage (Müller and Rieu, 2016). For instance, heat stress induces premature tapetum degeneration in *Arabidopsis thaliana* (Baron et al., 2012), rice (Ku et al., 2003), and wheat (Omidi et al., 2014). Conversely, cold stress disturbs tapetum development by delaying or inhibiting tapetal PCD (Liu et al., 2019). Various signaling pathways play pivotal roles in tapetum development and degeneration processes, including hormone and sugar signaling (Wang et al., 1999; Sun et al., 2019). Oliver et al. (2007) reported that in rice, male gametogenesis occurring under low-temperature stress, increased abscisic acid (ABA) levels and suppressed the expression of the tapetum-specific cell wall invertase (*CWIN*), *OsINV4*, and monosaccharide transporter genes, *OSMST8* and *OSMST7*. These expression changes interfere with tapetum PCD and lead to pollen abortion (Oliver et al., 2007).

As sessile organisms, plants have developed a variety of responses to tolerate environmental stress, including transcriptional, post-transcriptional, and post-translational regulatory processes (Barrero-Gil and Salinas, 2013; De Storme and Geelen, 2014; Guerra et al., 2015; Zhu, 2016). Post-transcriptional modifications based on RNA alternative splicing, processing, and modification shape the transcriptome in response to abiotic stress. While, N6-methyladenosine (m^6^A), a methylation at the N6 position of adenosine, is the most abundant internal mRNA modification, which plays an important role in post-transcriptional gene expression regulation (Yue et al., 2015) by affecting RNA splicing (Liu et al., 2015; Xiao et al., 2016), stability (Wang et al., 2014; Du et al., 2016), translation (Zhou et al., 2015), and export (Roundtree et al., 2017). m^6^A methylation is recognized by RNA-binding proteins (readers), and its levels are dynamically modified by RNA methyltransferases (Writers) and demethylases (Erasers) (Yue et al., 2019). Recent evidences demonstrated that m^6^A also participates in plant responses to various abiotic stresses. In *Arabidopsis*, changed m^6^A deposition affects RNA secondary structure under salt stress, resulting in increased stability of mRNA transcripts of abiotic stress response genes (Kramer et al., 2020). Additionally, *ALKBH6* (eraser) mutation results in increased salt, drought, and heat stress sensitivity during seed germination (Huong et al., 2020).

In *Arabidopsis*, m^6^A levels vary across tissues, with a high m^6^A/A ratio occurring in flowers (Zhong et al., 2008). MTA (one of the earliest discovered methyltransferases in *Arabidopsis*) disruption results in embryo development arrest at the globular stage (Zhong et al., 2008). Furthermore, the ALKBH10B demethylase (eraser) affects floral transition by regulating mRNA m^6^A of key flowering genes (Duan et al., 2017). In rice, OsFIP (writer) regulates early microspore degeneration during male gametogenesis (Zhang et al., 2019). The tomato ALKBH2 participates in fruit ripening by affecting the stability of a DNA demethylase gene, *SlDML2*, *via* m^6^A modification (Zhou et al., 2019). These findings suggest that m^6^A plays essential roles in regulating reproductive developmental processes. However, whether and how m^6^A participates in temperature stress response during the plant male reproductive stage remain elusive.

Tomato is one of the most widely cultivated subtropical vegetable crops in the world and its fruits and seeds are mainly derived from sexual reproduction. Because of its tropical origin, tomato is extremely sensitive to low temperature. In this study, we used m^6^A sequencing to analyze changes in m^6^A methylation in response to moderate low-temperature (MLT) stress during tomato anther development. We found that m^6^A methylation was prevalent in tomato anther mRNA but that the levels declined upon MLT stress. Furthermore, MLT stress directly affected m^6^A methylation abundance on a set of transcripts that regulate corresponding gene expression involved in anther development. Finally, we demonstrated that the decreased expression of an ATP-binding transcript, *SlABCG31*, was inversely correlated with its high m^6^A deposition, which resulted in high ABA in MLT-stressed anthers. The decrease in *SlABCG31* expression may also participate in aberrant pollen coat formation after low temperature exposure. Overall, we demonstrated that mRNA m^6^A modification was associated with anther development under low-temperature conditions.

## Materials and Methods

### Plant Material and Growth Conditions

The tomato (*Solanum lycopersicum*) cultivar “Micro-Tom” provided by the Tomato Genetics Resource Center (University of California, Davis), was used in all experiments. Plants were grown in controlled chambers with 25°C ± 1/20 ± 1°C (normal temperature, NT), 16/8-h light/dark cycles, 300 μmol photons m^–2^ s^–1^ light intensity, and 60%–70% relative humidity. Six-week-old flowering plants were exposed to a 10°C/10°C (day/night) MLT treatment for 6 days and then moved back to NT for recovery.

### Pollen Phenotypic Analysis and Light Microscopy

The development stages of the tomato anther are closely correlated to the flower bud size (Brukhin et al., 2003), which were roughly classified into six different stages (I–VI) according to semithin section observation as previously described (Peng et al., 2013; Chen et al., 2018). The anther at tetrad stage (stage II) were randomly marked with color strings before exposure to low temperature, because the tetrad stage is the most sensitive to ambient temperatures (Paupière et al., 2017; Begcy et al., 2019). The samples (anther at stages I–VI) harvested immediately after 6 days of MLT treatment were named MLT. Then the MLT-stressed plants were moved back to NT for recovery growth. The samples (anthers stages III–VI) developed from the marked tetrad anther at different time intervals that corresponded to time taken by tetrad anther to reach next different anther stages (stages III–VI) were named MLTR.

Mature pollen grain viability was determined using Alexander staining (Alexander, 1969). Scanning electron microscopy (SEM) was performed as previously described (Chen et al., 2018). Semithin section analysis was performed according to a previous report (Chen et al., 2018) with minor modifications. Briefly, the samples were fixed for 12 h at 4°C with 0.1 M phosphate-buffered saline (PBS) (pH 7.2) containing 2.5% glutaraldehyde (v/v). Subsequently, they were washed thrice with 0.1 M PBS, followed by soaking in 1% osmic acid (v/v) for 1–2 h. Specimens were rewashed thrice with 0.1 M PBS and then dehydrated through a gradient ethanol series. Further, we embedded and polymerized the specimens in Spurr’s resin, and cut them into 2-μm-thick sections, which were stained with 1% methylene blue. Sections were observed and photographed under a Nikon Eclipse 90i microscope (Nikon, Japan). Terminal deoxynucleotidyl transferase-mediated dUTP nick-end labeling (TUNEL) assays were performed with the TUNEL Apoptosis Detection Kit (Roche, Switzerland) according to the manufacturer’s protocol and using 10-μm paraffin sections of anthers at different stages. Furthermore, fluorescence signals were analyzed using Nikon’s confocal laser scanning microscope, A1-SHS (Nikon, Japan).

### RNA Isolation and qRT-PCR Analyses

Immediately after 6 days of MLT stress, anthers (about 0.5 g, >15 plants for each sample) at the six different stages (I–VI) were manually sampled and subjected to total RNA isolation using the Total RNA Kit II (OMEGA, United States). First-strand cDNA was synthesized using the PrimeScript^TM^ RT reagent kit (Takara, Japan). Quantitative RT-PCR reactions were performed using SYBR^®^ Green Realtime PCR Master Mix (Toyobo, Japan). Reactions were run on Bio-Rad CFX96 (Bio-Rad, United States) in triplicate technical replicates and with *SlUbi3* as the endogenous control. Ct values were recorded and normalized with the 2^–ΔΔ*Ct*^ method (Livak and Schmittgen, 2001). The experiment was repeated as three independent biological replicates. Primers used for qRT-PCR analysis are listed in Supplementary Table 1.

### Quantitative Analysis of m^6^A in Total RNA

The total RNA m^6^A levels were determined with the EpiQuik^TM^ m^6^A RNA Methylation Quantification Kit (Colorimetric) (Epigentek, United States) following the manufacturer’s instructions. Briefly, RNA was bound to strip wells using RNA high binding solution. RNA m^6^A modification was captured and detected with capture and detection antibodies. Signals were enhanced and quantified colorimetrically by determining the absorbance at 450 nm using a microplate spectrophotometer with three technical replicates. Both negative and positive control RNA samples (m^6^A 2 μg/mL) were provided with the kit. The standard curve was generated using the positive control sample. All experiments were performed three independent times.

### High-Throughput m^6^A Sequencing

m^6^A-seq was performed as previously described (Dominissini et al., 2013). The anthers (about 0.5 g) at the tetrad stage from NT- and MLT-treated plants (*n* > 15 plants for each sample) were collected to extract the total RNAs. Three biological replicates of m6A RIP sequencing were performed. RNA quality was checked with the Agilent 2100 bioanalyzer (Agilent, United States) and using gel electrophoresis. Separation of mRNAs from total RNAs and sequencing of immunoprecipitated mRNA and pre-immunoprecipitated mRNA (input control) was performed at Novogene Biotech (Beijing, China) using an Illumina HiSeq 4000 system. Sequence data were deposited into the NCBI Sequence Read Archive under accession BioProject ID: PRJAN713408. A total of 23–29 million clean reads were produced from each library, and more than 95% of the reads were uniquely mapped to the tomato reference genome (Tomato Genome Consortium, 2012). The MACS software was used to identify m^6^A peaks in immunoprecipitated samples using the corresponding input sample as the control. The strict cutoff criterion with assigned false discovery rate < 0.05 was applied to obtain high-confidence peaks (Zhang et al., 2008). Only peaks captured in all three biological samples were considered as confident peaks and used for further analysis. The *de novo* motif identification of the m6A peak data was carried out using the HOMER (hypergeometric optimization of motif enrichment) software (version 4.7^1^) to obtain their position and accurate motif regions. Visualization analysis of m^6^A peaks was performed using the Integrated Genome Browser (Helt et al., 2009). All m^6^A modification sites were assigned to different transcription regions covering transcription start site (TSS), 5′ untranslated region (UTR), coding sequence (CDS), 3′UTR, and intron. The gene expression level was calculated using RPKM method (Reads Per Kilobase per Million mapped reads). Differential m^6^A peaks between NT and MLT anthers were determined using the m^6^A site differential algorithm (Meng et al., 2013) with a *P*-value < 0.05. Gene Ontology (GO) and Kyoto Encyclopedia of Genes and Genomes (KEGG) enrichment analysis of were performed to identify the biological processes involving the differentially modified genes.

### Quantification of Endogenous ABA Levels

Measurement of endogenous ABA was performed as previously reported (Chen et al., 2018) with minor modifications. Fresh anthers (about 0.5 g) at the tetrad stage were collected from NT and MLT plants (>15 plants for each sample) and ground into a powder in liquid nitrogen with a mortar and pestle. For each sample, 100 mg of powder was homogenized using 1 mL ethyl acetate spiked with d6-ABA (OlchemIm, Czechia). Samples were then agitated for 10 min and centrifuged at 13,000 *g* for 20 min at 4°C. The supernatant was collected and dried in N_2_ gas. Dried samples were resuspended in 0.2 mL of 60% (v/v) methanol and then centrifuged at 13,000 *g* for 10 min at 4°C. The supernatant sample was filtered through a 0.22-μm nylon membrane and analyzed using HPLC/MS-MS on an Agilent 1290 Infinity HPLC system coupled with an Agilent 6460 Triple Quad LC/MS device (Agilent, United States). Agilent Zorbax XDB C18 columns (150 mm × 2.1 mm, 3.5 μm) were used to perform the HPLC analysis. Three independent biological replicates were performed for each sample.

### Statistical Analysis

All experiments were conducted in randomized complete block design. Mean values were used to represent the data of at least three independent experiments. The statistical differences between treatments were verified using two-tailed Student’s *t*-tests.

## Results

### Impact of MLT Stress on Tomato Pollen Development

**FIGURE 1:**
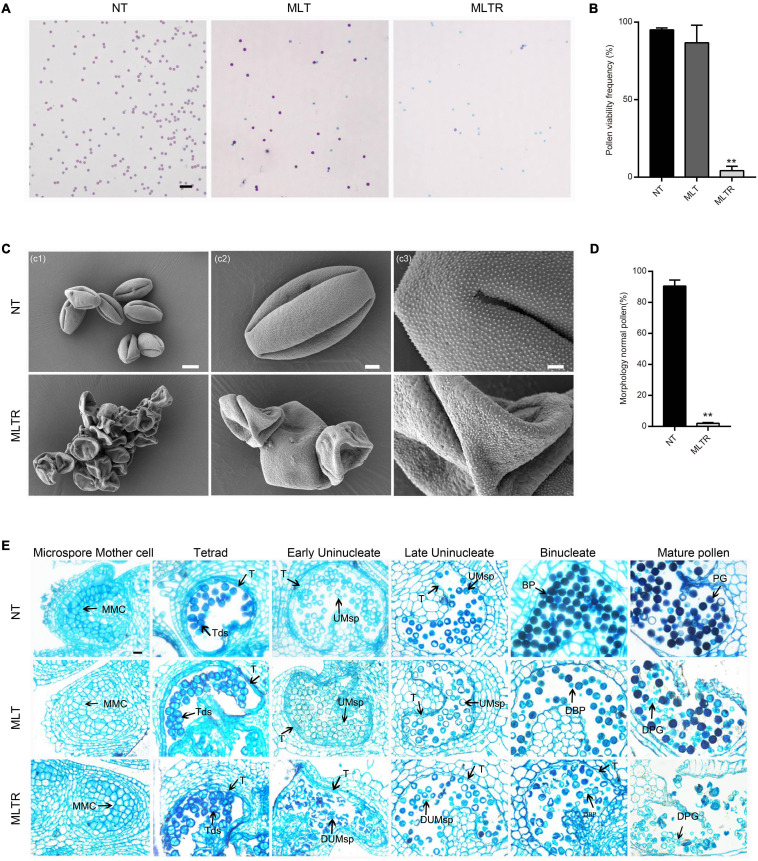
Moderate low temperature (MTL) stress at the tetrad stage affects pollen development in tomato. **(A)** Alexander staining of mature pollen grains from the plants exposed to normal temperature (NT), MLT-stress and returned to normal growing temperature for recovery after moderate low temperature treatment (MLTR). Scale bars = 100 μm. **(C)** Scanning electron microscopy indicated that MLTR-stress pollen grains were seriously shrunk and collapsed, appearing aggregated. Scale bars c1 = 10 μm; c2 = 3 μm; c3 = 1 μm. **(B,D)** Quantification of viable mature pollen grains and morphology normal pollen from NT, MLT, and MLTR plants, respectively. Each value is the mean ± SD (*n* = at least three biological replicates with 15 plants each). *P < 0.05; **P < 0.01 (two-tailed Student’s *t-* test). **(E)** Semithin cross sections of anthers in different stages under NT and MLT condition. The tapetum degeneration was postponed under MLTR compare to NT condition. MMC, microspore mother cell; Tds, tetrads; UMsp, uninucleate microspore; BP, binucleate pollen; PG, pollen grains; DUMsp, degenerated uninucleate microspore; DBP, degenerated binucleate pollen; DPG, degenerated pollen grains; T, tapetum. The experiment was repeated three times independently with consistent results. Scale bars = 20 μm.

To investigate tomato pollen development response to low-temperature stress, flowering tomato plants were treated with MLT (10°C/10°C) for 6 days and then moved to normal growing conditions for recovery. Plants continuously grown under NT (25°C/20°C) were used as controls (Supplementary Figure 1A). Surprisingly, after 6 days of MLT treatment, pollen viability was similar in control and stressed plants, reaching 95.0% (Figures 1A,B). Tomato reproductive organ development nearly ceases when the ambient temperature is below 12°C (Criddle et al., 1997). Therefore, the anther development may be delayed and cease under MLT-stress. We determined the viability of mature pollen grains developed from marked anthers at the tetrad stage after MLTR. Strikingly, pollen viability decreased by 95.8% after MLTR (Figures 1A,B). We also examined pollen morphology using SEM. Under NT, pollen had an elliptical shape and grain surface was densely microperforated and granulated with three germination ditches (Figure 1C). Conversely, 97.9% of MLTR-stressed pollen grains were highly shriveled and aggregated. The pollen grain surface was smooth, lacking granulation and germination ditch (Figures 1C,D). To monitor the dynamic response to MLT stress, we generated transverse semithin sections of anthers at different developmental stages and assessed their morphology. Under NT, tapetal cells formed a regular layer surrounding the locules at tetrad stage, which subsequently underwent PCD, gradually degenerating until disappearing completely at binucleate stage (Figure 1E). Anther morphology did not differ between MLT and NT plants, except for few pollen grain abortions at mature pollen stage (Figure 1E). However, under MLTR-stress, at early uninucleate stage, anther chambers were irregular and harbored numerous shriveled microspores, and tapetal cells became vacuolated. More importantly, tapetal cell degeneration was incomplete even at the binucleate stage, contributing to pollen grain abortion at the mature stage (Figure 1E). To further investigate the timing of tapetal cell degeneration in pollen development, we performed TUNEL assays on different stage anthers from plants grown under NT or MLTR conditions. Under NT, TUNEL signals were first detected on tapetal cells at the tetrad stage. They gradually intensified from the early to late uninucleate stage and finally disappeared at the binucleate stage (Supplementary Figure 1B). However, under MLTR, TUNEL signals appeared at the uninucleate stage and became weaker at the binucleate stage, suggesting that tapetal cells PCD was delayed (Supplementary Figure 1B). Our data show that pollen development was sensitive to low temperature and that plants exposed to MLT stress had pollen sterility, characterized by changes in tapetum development and exine deposition.

### MLT Stress Influences m^6^A Levels and m^6^A-Related Gene Expression in Tomato Anthers

**FIGURE 2:**
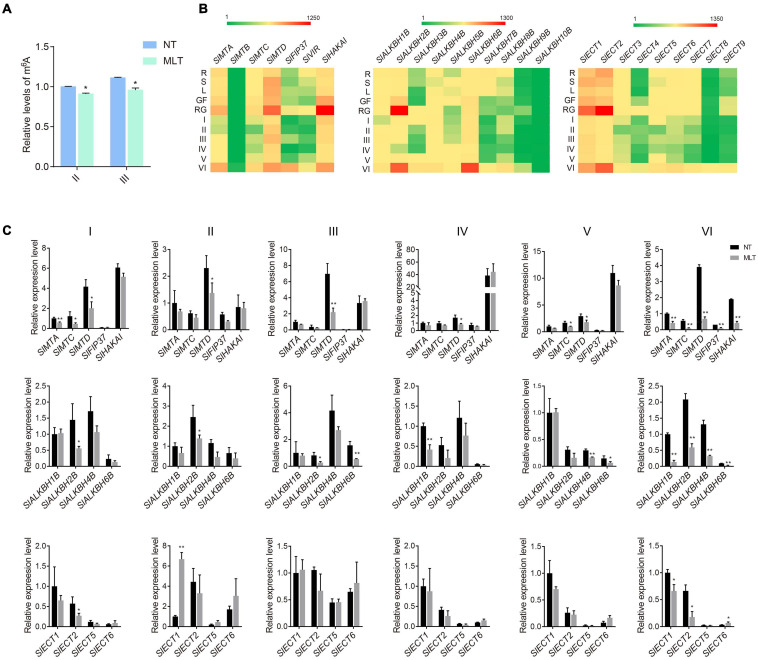
The expression profiles of m^6^A writers, erasers and readers under NT and MLT conditions. **(A)** The relative levels of m^6^A in total RNA of anther at stage-II and III under NT and MLT conditions. The level of m^6^A in stage-II under NT condition is set to 1. Each value is the mean ± SD (*n* = at least three biological replicates with 15 plants each). *P < 0.05; **P < 0.01 (two-tailed Student’s *t-* test). **(B)** Expression patterns of genes in various tissues of plants under NT condition, especially in anther of different stages. Green, weak expression; Red, strong expression. R, root; S, stem; L, leaf; GF, green fruit; RF, red fruit; I, anther at microspore mother cell stage; II, anther at tetrad stage; III, anther at early uninucleate stage; IV, anther at late uninucleate stage; V, anther at binucleate stage; VI, anther at mature pollen stage. **(C)** qRT-PCR analysis of writer, erasers and readers genes in anther at different stages under NT and MLT conditions. The levels of genes expression normalized to *Ubiquitin* expression are shown relative to *SlMTA*, *SlALKBH1B*, and *SlECT1* under NT condition set to 1, respectively. Each value is the mean ± SD (*n* = at least three biological replicates with 15 plants each). *P < 0.05; **P < 0.01 (two-tailed Student’s *t-* test).

To determine whether m^6^A participates in tomato anther development and responds to suboptimal temperature stress, we performed colorimetric m^6^A quantification assays in plants grown under NT and MLT. Under NT conditions, overall RNA m^6^A levels were slightly lower at the tetrad stage than at the pollen mature cells (PMC) stage. m^6^A increased mildly at the uninucleate stage and peaked at the binucleate stage (Supplementary Figure 2A). However, under MLT stress, anther m^6^A levels were decreased at both the tetrad and uninucleate stages compared to that at NT (Figure 2A).

Next, we analyzed the spatial and temporal expression profiles of m^6^A-related components (writers, erasers, and readers) in NT tomato plants using qRT-PCR. Among the 26 investigated genes (seven writers, ten erasers, and nine readers), 23 had altered expression among different tissues or anthers at different developmental stages. The exceptions were *SlMTB*, *SlALKBH9B*, and *SlALKBH10B*, which had low expression levels in all 11 tested organs/tissues. The writer genes, including *SlMTA*, *SlMTD*, and *SlHAKAI*, were mostly expressed in tomato anthers at the mature pollen stage (VI) and fruits (Figure 2B). Several erasers, including *SlALKBH2B* and *SlALKBH6B*, were preferentially expressed in mature anthers and young fruits, whereas *SlALKBH7B* and *SlALKBH8B* were rarely expressed in reproductive tissues (Figure 2B). Moreover, *SlECT1* and *SlECT2* reader genes were also preferentially expressed in mature anthers and young fruits; they had higher expression levels than other readers, including *SlECT3*, *SlECT4*, and *SlECT7*, which had low expression in anthers at all stages except stage VI (Figure 2B). The expression profiles of m^6^A-related genes analyzed using qRT-PCR assay were consistent with the *in silico* data available from the tomato eFP Browser (Supplementary Figures 2B–D).

Since total RNA m^6^A levels were lower in MLT anthers at stages II and III, we further analyzed the changes in m^6^A-related gene expression profiles in MLT and NT anthers using qRT-PCR. *SlMTA*, *SlMTC*, and *SlMTD* expression significantly decreased at the microspore mother cell stage (I) under MLT stress compared with that at NT. At tetrad (II) and uninucleate (III and IV) stages, only *SlMTD* expression significantly decreased (Figure 2C), whereas *SlMTC* and *SlMTD* were significantly downregulated at the binucleate stage (V) under MLT stress. More importantly, all writer genes analyzed (*SlMTA*, *SlMTC*, *SlMTD*, *SlFIP37*, and *SlHAKAI*) were downregulated in MLT anthers at the mature pollen stage (VI) (Figure 2C). Furthermore, the *SlALKBH1B* eraser gene was significantly downregulated in anthers at the stages IV and VI. *SlALKBH2B* was significantly downregulated at stages II, III, and VI, and *SlALKBH4B* and *SlALKBH6B* were decreased at both stages V and VI (Figure 2C). However, the m^6^A reader *SlECT1* was significantly upregulated in MLT at stage II (Figure 2C). These data indicate that m^6^A modification levels and the expression pattern of its related genes were changed under low-temperature stress. We speculated that m^6^A methylation plays a fundamental role in anther development and temperature stress responses.

### Transcriptome-Wide Detection of m^6^A Methylation in Tomato Anthers

**FIGURE 3:**
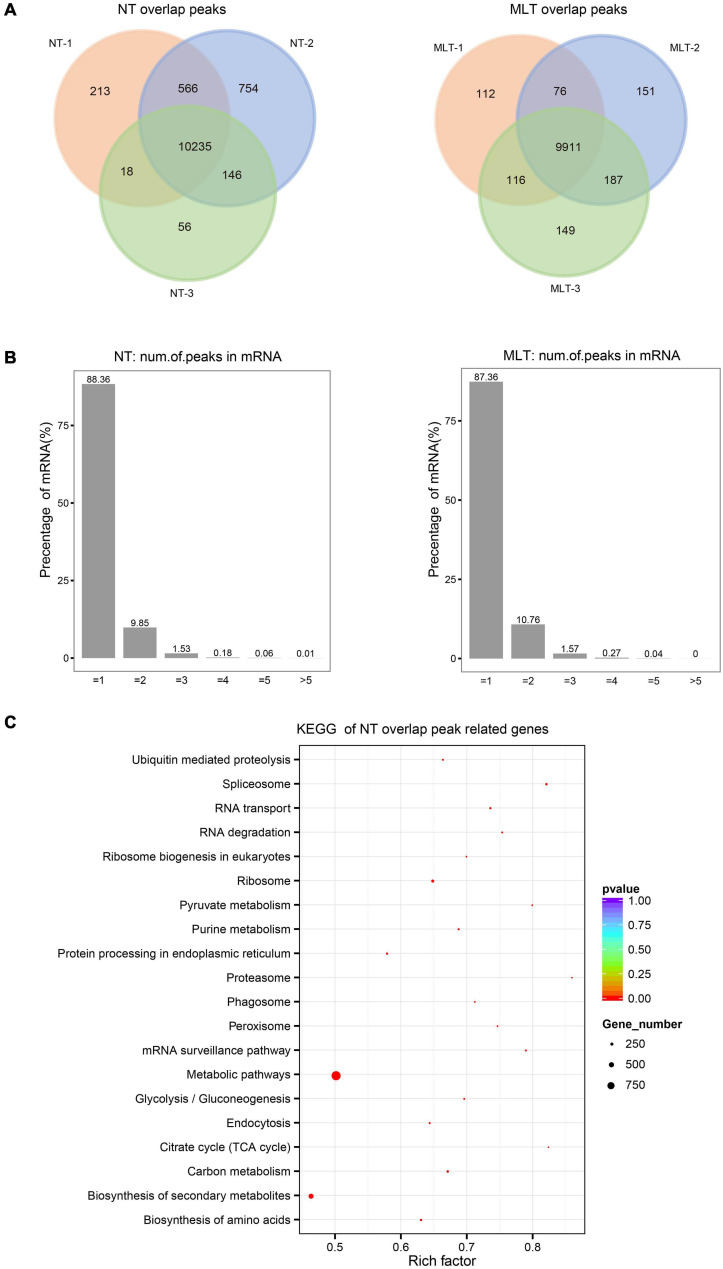
Transcriptome-wide m^6^A methylation profiles in tomato anther at stage-II. **(A)** The number of overlapped m^6^A peaks identified in three biological replicates under NT and MLT conditions, respectively. The peaks identified in all three biological replicates were used for subsequent analysis. **(B)** Statistics on proportion of the m^6^A-modifited transcripts containing different m^6^A sites numbers in NT and MLT conditions. **(C)** KEGG enrichment analysis of all the transcripts with m^6^A peaks in NT condition.

To explore the molecular mechanisms behind m^6^A regulation of anther development in tomato, we performed a transcriptome-wide detection of m^6^A methylation through m^6^A-seq using NT- and MLT- anthers at the tetrad stage. Six MeRIP libraries were analyzed (Supplementary Figure 3A) and high Pearson correlation coefficients were found between biological replicates, indicating a high reproducibility for the m^6^A-seq data (Supplementary Figure 3B). m^6^A modification sites, named m^6^A peaks, were identified on the basis of the comparison of read distribution between the input and immunoprecipitation samples using exomePeak with an estimated *P*-value < 0.05. We used high-confidence m^6^A peaks that were observed in all three biological replicates for subsequent analysis. A total of 10,235 and 9,911 high-confidence m^6^A peaks, representing 8,541 and 8,743 gene transcripts, were detected in NT and MLT anthers, respectively (Figure 3A and Supplementary Figure 3C). Among transcripts harboring m^6^A modification, 88.36, 9.85, 1.53, and 0.26% contained one, two, three, and more than three peaks, respectively (Figure 3B). Enrichment analysis using the KEGG revealed that these m^6^A-containing transcripts in NT condition were enriched in multiple conserved metabolic pathways, including tricarboxylic acid (TCA) cycle, spliceosome, proteasome, and pyruvate metabolism (Figure 3C).

**FIGURE 4:**
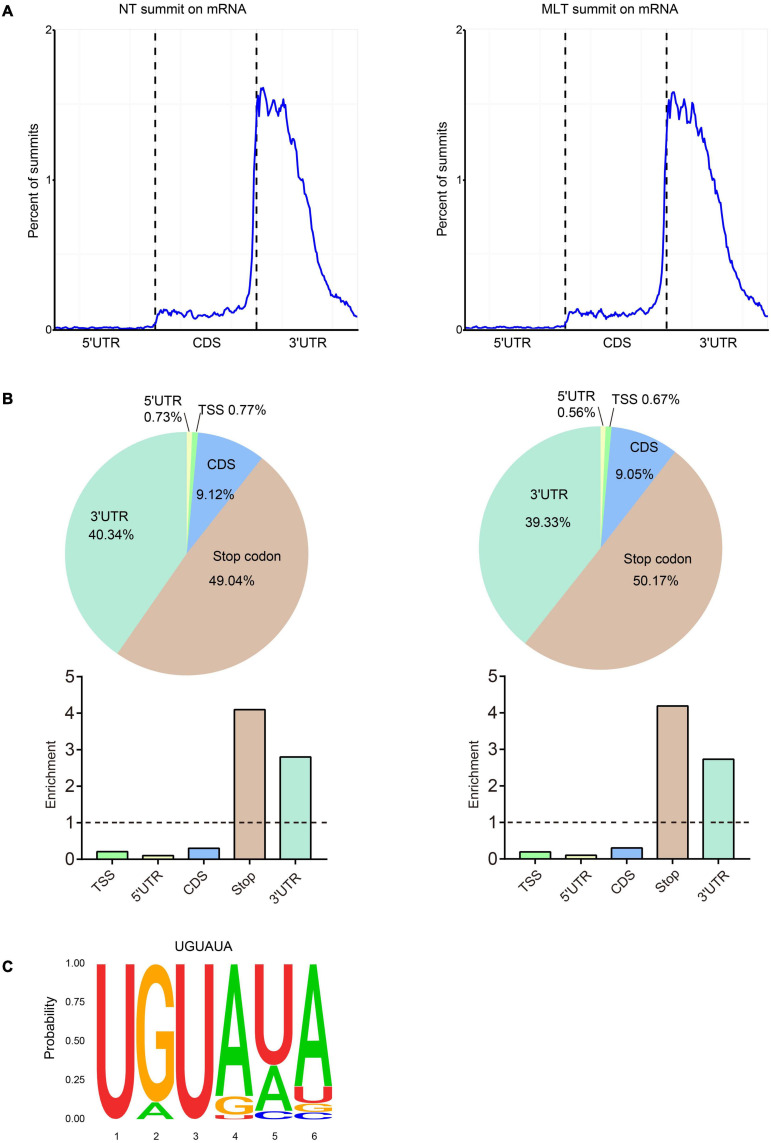
Distribution pattern of m^6^A peaks along the transcripts and the sequence motif in tomato anther. **(A)** m^6^A peaks summits localization along the transcripts in NT and MLT conditions. Each transcript is divided into three parts: 5′-UTR, CDS and 3′ -UTR. UTR, untranslated region; CDS, coding sequence. **(B)** The m^6^A peaks summits and relative enrichment in five non-overlapping transcripts segments in NT and MLT conditions. TSS, transcription start site. **(C)** The conserved sequence motif for m^6^A peaks in NT and MLT conditions.

The analyses of m^6^A peak distribution across the tomato transcriptome in NT anthers showed that highly enriched m^6^A modifications surrounded stop codon and 3′-UTRs (Figure 4A), as seen in tomato fruits (Zhou et al., 2019). Furthermore, we assigned each m^6^A peak to one of five transcript segments based on their location: TSS, 5′-UTR, CDS, stop codon (a 100-nucleotide window centered on the stop codon), and 3′-UTR. These analyses revealed that m^6^A peaks were abundant near stop codons (49.04%) and 3′-UTRs (40.34%), followed by CDS (9.12%) (Figure 4B). After segment normalization by the total length of each gene portion, we confirmed that m^6^A peaks were predominantly enriched around the stop codon and 3′-UTR (Figure 4B). Homer was used to identify the sequence motifs enriched within the m^6^A peaks in tomato anthers and it showed that UGUAYY (where Y represents A, G, U, or C) was the most statistically over-represented motif (Figure 4C). This phenomenon was observed previously in *Arabidopsis* and tomato fruits (Luo et al., 2014; Zhou et al., 2019), suggesting that m^6^A methylation sequence motifs are conserved between *Arabidopsis* and tomato.

### Comparison Between NT and MLT Anther m^6^A Methylation Profiles

**FIGURE 5:**
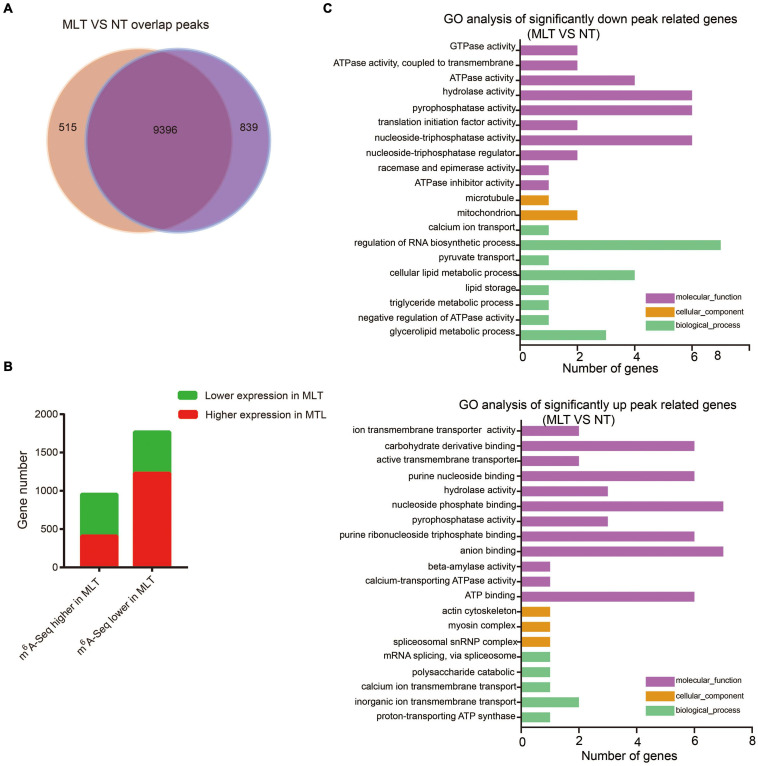
Comparative analysis transcriptome-wide m^6^A profiles between NT and MLT anthers at stage-II. **(A)** The number of overlapped m^6^A peaks between NT and MLT anthers. **(B)** The gene number of transcripts with differential m^6^A enrichment and differential transcript levels between NT and MLT anthers. **(C)** GO analysis significantly different enrichment peaks related genes between NT and MLT anthers.

To systemically elucidate the m^6^A methylome response to low temperature, we compared NT and MLT m^6^A-seq data. There was no obvious difference in whole transcriptome m^6^A distribution between NT and MLT anthers (Figure 4A). The analysis showed that 7,846 m^6^A modified transcripts containing 9,396 m^6^A peaks were shared by MLT and NT (Figure 5A and Supplementary Figure 3D). However, a total of 3,837 m^6^A peaks harboring 3,434 gene transcripts differed between NT and MLT anthers in m^6^A levels (*P*-value < 0.05) (Supplementary Table 2). Among them, the m^6^A peaks of 2,783 transcripts appeared in stop codons or 3′-UTRs; the m^6^A levels of 978 of these transcripts were higher in MLT than in NT, whereas the inverse was true for 1,805 transcripts (Figure 5B and Supplementary Table 2). These data suggested that MLT stress markedly affected m^6^A levels in a large number of mRNA transcripts, which are predominantly downregulated.

To identify the potential correlation between m^6^A levels and transcript abundance in tomato anthers, we investigated the transcript levels of genes under MLT stress and NT by RNA-seq. Among the 1,805 transcripts with lower m^6^A levels in MLT anthers, 70.2% were upregulated under MLT stress, whereas 29.8% were downregulated. Accordingly, among 978 transcripts with higher m^6^A levels in MLT anthers, 569 had lower expression levels in MLT-stressed anthers (Figure 5B). These results revealed that m^6^A methylome in stop codon or 3′-UTR generally negatively correlated with the transcript levels. KEGG analysis showed that, under MLT stress, transcripts with increased m^6^A methylation were significantly enriched in phenylalanine, tyrosine, and tryptophan metabolism and amino acid biosynthesis pathways. Furthermore, transcripts with decreased m^6^A were significantly enriched in mRNAs for surveillance and carbon metabolism pathways (Supplementary Figure 4). These metabolic pathways may be associated with plant pollen development (Yang et al., 2001; Min et al., 2014; Fang et al., 2016).

### MLT Stress Induces the m^6^A Level Alteration in Anther-Related Transcripts

To further analyze m^6^A response to MLT stress during anther development, we performed GO analysis of the transcripts that were significantly differentially modified by m^6^A methylation (| log_2_FoldChange| > 1 and *P*-value < 0.05) between NT and MLT stress. In MLT anthers, transcripts with decreased m^6^A methylation were significantly enriched in “cellular lipid metabolic process,” “regulation of RNA biosynthetic process,” and “adenosine triphosphatase (ATPase) activity-related function” categories (Figure 5C). Whereas the transcripts with increased m^6^A methylation under MLT stress were significantly enriched in “carbohydrate derivative binding,” “nucleoside phosphate binding,” “anion binding,” and “ATP-binding” categories (Figure 5C). These changed pathways play important roles in anther development (Lucca and León, 2012; Shi et al., 2015; Zhao et al., 2016).

**FIGURE 6:**
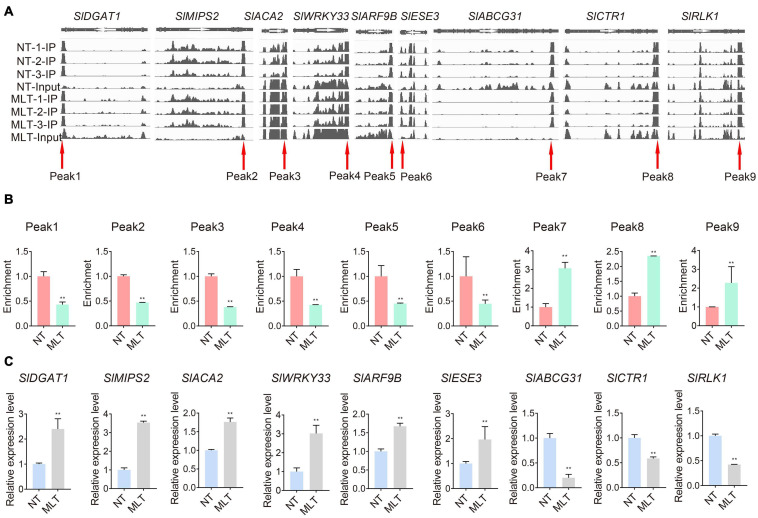
Comparison on m^6^A levels and expression levels of transcripts in NT and MLT anthers at stage-II. **(A)** Integrative Genome Browser (IGB) showing m^6^A-seq read distributions in transcripts. The red arrowheads indicate the position of m^6^A peaks with significantly changed m^6^A enrichment in MLT-stress stage-II anther compared to NT. The white arrowheads represent the direction of gene. **(B)** The m^6^A enrichment of transcripts involved in pollen development in NT and MLT anthers. The level of m^6^A in stage-II under NT condition is set to 1. Each value is the mean ± SD (*n* = 3 biological replicates with 15 plants each). *P < 0.05; **P < 0.01 (two-tailed Student’s *t-* test). **(C)** qRT-PCR analysis validates the different m^6^A enrichment peak related genes expression levels between NT and MLT anthers. The levels of genes expression normalized to *Ubiquitin* expression are shown relative to genes expression under NT condition set to 1, Each value is the mean ± SD (*n* = at least three biological replicates with 15 plants each). *P < 0.05; **P < 0.01 (two-tailed Student’s *t-* test).

We further analyzed the expression patterns of the significantly differentially modified transcripts in response to MLT stress. The transcripts of diacylglycerol acyltransferase 1 (*SlDGAT1*) and inositol-3-phosphate synthase 2 (*SlMIPS2*), which are involved in lipid metabolism, were significantly upregulated under MLT stress and had decreased m^6^A levels (Figure 6). Conversely, calcium-transporting ATPase isoform 2 (*SlACA2*) expression was downregulated under MLT stress (Figure 6C). The transcript levels of factors with decreased m^6^A modification, including WRKY DNA-binding protein 33 (*SlWRKY33*), auxin response factor 9B (*SlARF9B*), and ethylene response factor 3 (*SlESE3*), were significantly upregulated in MLT anthers (Figure 6). Additionally, the expression of the ATP-binding member of ATP-binding cassette G31 (*SlABCG31*), CTR1-like protein kinase (*SlCTR1*), and receptor-like kinase 1 (*SlRLK1*) genes, which had high m^6^A methylation levels, was downregulated under MLT stress (Figure 6C). Overall, these data implied that MLT stress affected m^6^A methylation of various transcripts, altering their expression levels to regulate pollen development.

### MLT Stress Increases *SlABCG31* m^6^A Levels and ABA Content

**FIGURE 7:**
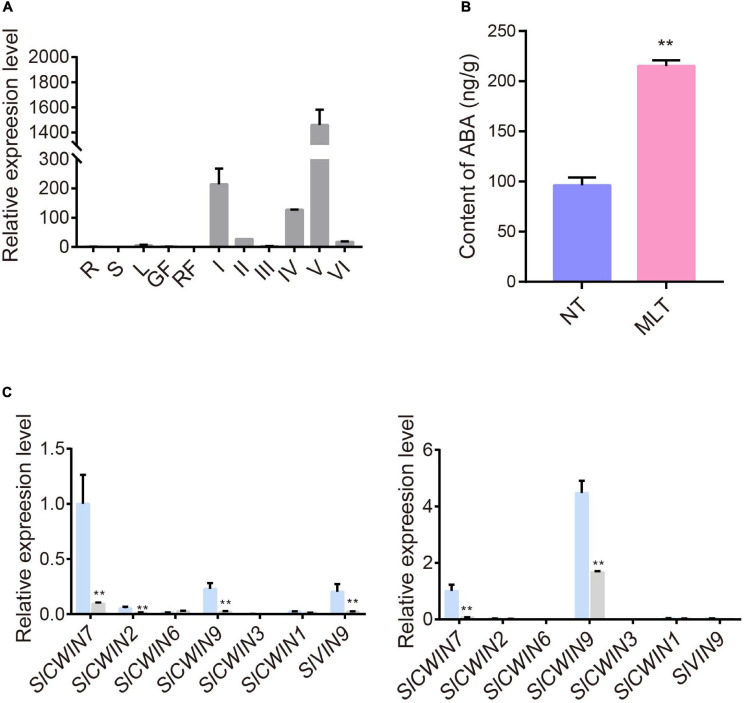
The expression profiles of *SlABCG31* and ABA levels in anther under NT and MLT conditions. **(A)** qPCR analysis of *SlABCG31* in various tissues of tomato, especially in anther at different stages. The genes expression levels normalized to *Ubiquitin* expression are shown relative to root set to 1. R, root; S, stem; L, leaf; GF, green fruit; RF, red fruit; I, anther at microspore mother cell stage; II, anther at tetrad stage; III, anther at early uninucleate stage; IV, anther at late uninucleate stage; V, anther at binucleate stage; VI, anther at mature pollen stage. Each value is the mean ± SD (*n* = at least three biological replicates with 15 plants each). **(B)** ABA levels in anther at stage-II under MLT-stress were increased in comparison to NT condition. Each value is the mean ± SD (*n* = at least three biological replicates with 15 plants each). *P < 0.05; **P < 0.01 (two-tailed Student’s *t-* test). **(C)** The expression levels of *SlCWINs* genes in anther at stage-II and III in NT and MLT conditions. The levels of genes expression normalized to *Ubiquitin* expression are shown relative to the expression of *SlCWIN7* in NT condition set to 1. Each value is the mean ± SD (*n* = at least three biological replicates with 15 plants each). *P < 0.05; **P < 0.01 (two-tailed Student’s *t-* test).

Because m^6^A levels changed in transcripts that may be involved in pollen development under MLT stress, such as *SlDGAT1*, *SlMIPS2*, *SlACA2*, and *SlABCG31* (Figures 5C, 6), we further examined the underlying mechanisms of pollen development regulation by m^6^A methylation. Members of the ATP-binding cassette transporter (ABC) family, particularly ABC sub-family G members, are involved in ABA transport. Accordingly, *AtABCG31* functions in exporting ABA from the endosperm to the embryo in *Arabidopsis* (Borghi et al., 2015; Kang et al., 2015). The tomato *SlABCG31* is highly homologous (71%) to *AtABCG31* (Supplementary Figure 5). To identify whether *SlABCG31* participates in pollen development under MLT stress, we analyzed its expression with qRT-PCR in different tomato tissues and anthers at different developmental stages. The data revealed that *SlABCG31* is predominantly accumulated in anthers and is rarely detected in other tissues and organs (Figure 7A). Because *SlABCG31* expression sharply decreased under MLT stress (Figure 6B), we investigated whether it affected ABA levels in MLT anthers. Surprisingly, endogenous ABA levels measured using HPLC–mass spectrometry were noticeably increased in MLT anthers than in NT anthers at the tetrad stage (Figure 7B). Furthermore, qRT-PCR data revealed that the expression of the ABA signaling-related genes, *SlPYLs* and *SlPP2Cs*, was significantly changed under MLT stress (Supplementary Figure 6). *SlPYL4*, which had the highest expression level at the tetrad stage, was significantly decreased in MLT anthers. *SlPP2C1* and *SlPP2C3* were significantly upregulated under MLT stress compared with those under NT condition.

**FIGURE 8:**
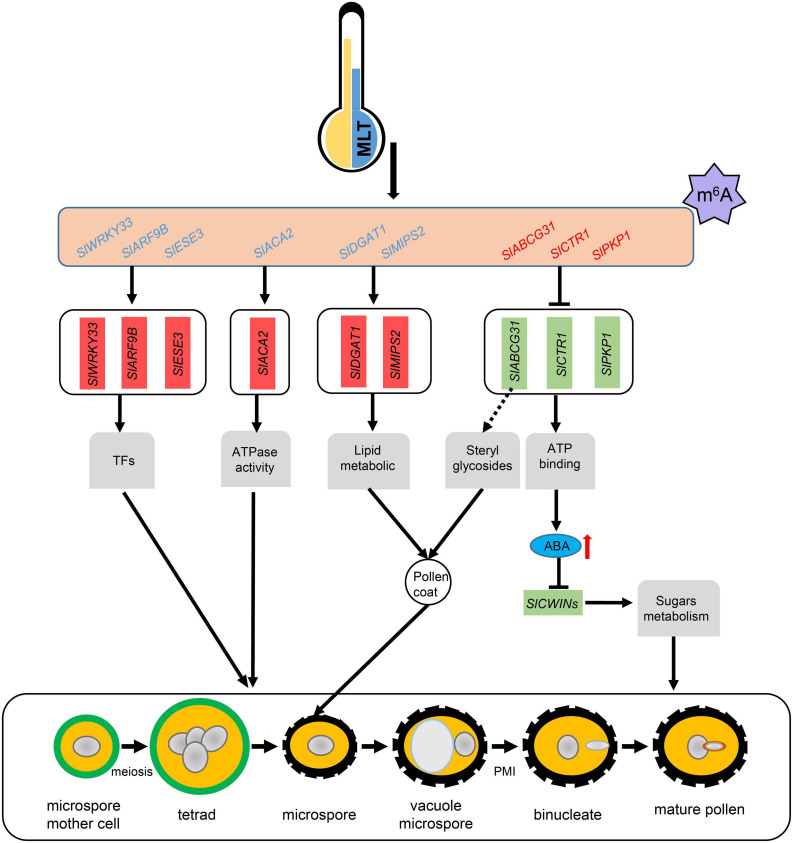
A working model for m^6^A methylation affects pollen development under MLT-stress. MLT, moderate low temperature; TFs, transcript factors; Blue letters means transcripts with lower m^6^A levels, and red letters means transcripts with higher m^6^A levels; Red and green boxes represent unregulated and downregulated genes expression levels, respectively; Red arrow, significant increase.

Increased ABA levels lead to high pollen abortion through negative regulation of *CWIN* expression, which may distort the anther carbohydrate pool (Oliver et al., 2007). Our qRT-PCR analysis showed that *SlCWIN7* and *SlCWIN9* were strikingly downregulated at the uninucleate stage under MLT stress (Figure 7C). Overall, we speculate that MLT stress increased m^6^A methylation in *SlABCG31*, resulting in its decreased expression, which in turn increased the ABA content in tomato anthers to suppress *SlCWINs* expression and cause pollen abortion (Figure 8).

## Discussion

N6-methyladenosine methylation is the most prevalent type of internal mRNA modification in eukaryotes. Previous studies revealed that m^6^A modification plays essential roles in embryo development, shoot stem cell fate, root development, and floral transition in *Arabidopsis* (Vespa et al., 2004; Zhong et al., 2008; Bodi et al., 2012; Růžička et al., 2017). In tomato, m^6^A methylation participates in fruit ripening regulation through *SlALKBH2*, which mediates the m^6^A methylation of *SlDML2* transcripts (Zhou et al., 2019). Recent study revealed that the OsFIP methylase may regulate early microspore development in rice (Zhang et al., 2019). Although the roles of m^6^A in plant growth and development have been demonstrated, mostly in *Arabidopsis*, its involvement and putative functions in the response to abiotic stress are still unknown. Here RNA methylome analysis revealed that mRNA m^6^A methylation is widespread in tomato anthers and is disturbed in response to low-temperature stress. Changes in m^6^A methylation may affect the expression of the genes involved in tapetum and microspore development through multiple pathways, at least partly through modifying ABA signaling genes (Figure 8).

Under normal growing conditions, anthers at the tetrad stage take about 6–7 days to reach the mature pollen stage. However, the reproductive development may slow down after exposure to low temperature stress. In our study, after 6 days MLT-stress, the marked anthers were still at tetrad stage, suggesting that at 10°C cold stress, the tomato anthers development nearly ceases, which is consistent with previous study showing that tomato reproductive organ development ceases when the ambient temperature is below 12°C (Criddle et al., 1997). Therefore, it is not surprising that the viability of mature pollen grains harvested immediately after 6 days (MLT) was similar to that in NT conditions. However, when the plants were moved back to NT conditions for recovery after cold stress, the marked anthers need another 6–7 days to reach the mature pollen stage (MLTR), and the mature pollen grains exhibited highly aborted (Figure 1E). Our cytological observations showed that low-temperature-induced pollen abortion could be attributed to delayed tapetal cell degradation at tetrad and early uninucleate stages, resulting in impaired pollen wall formation and pollen abortion (Figure 1). Correspondingly, m^6^A levels in total RNA were significantly decreased in anthers at both tetrad and uninucleate stages after MLT exposure (Figure 2A). Furthermore, *SlMTD* expression was significantly decreased at tetrad and early uninucleate stages, whereas *SlECT2* was dramatically overexpressed at the tetrad stage under MLT stress (Figure 2C). m^6^A-seq analysis revealed that MLT treatments significantly altered the m^6^A levels of 2,783 transcripts in tomato anthers at the tetrad stage, which may contribute to abnormal pollen development. These data indicated that m^6^A modification might be involved in tomato anther development under MLT stress. Among the altered transcription, 65% were downregulated. Similarly, in rice, knockouts of the methylase genes, *OsFIP* or *OsMTA2*, dramatically reduced m^6^A levels and resulted in microspore degeneration at the uninucleate stage and in a male sterility phenotype (Zhang et al., 2019). These results support the speculation that m^6^A methylation participates in male developmental regulation. However, our findings firstly reveal that m^6^A methylation may be involved in low-temperature stress responses during anther development.

Growing evidence suggests that diverse abiotic stresses can change transcript m^6^A methylation levels (Hu et al., 2019; Huong et al., 2020; Liu et al., 2020). Moreover, m^6^A levels usually negatively regulate gene expression by influencing mRNA stability and the subsequent protein synthesis process (Shen et al., 2016; Duan et al., 2017; Zhao et al., 2017). By combining MeRIP-seq and RNA-seq analyses on NT and MLT anthers at the tetrad stage, we revealed that, at the transcriptome-wide level, transcript m^6^A enrichment was usually inversely correlated with gene expression levels under MLT stress (Figure 5B). Some of these transcripts are involved in lipid metabolism, ATPase activity, and ATP-binding processes and these pathways have been well characterized in plant male organ reproduction (Figures 5C, 6; Lucca and León, 2012; Shi et al., 2015; Zhao et al., 2016). These results suggest that changes in m^6^A methylation under MLT stress may affect target mRNA abundance, ultimately leading to pollen abortion. Several studies revealed abiotic stress causes transcriptome-wide redistribution of the m^6^A modification. For example, heat shock increases m^6^A enrichment at 5′-UTRs, and it mainly mapped to regions surrounding stop codons in NT condition. The m^6^A modification at 5′-UTR promotes mRNA cap-independent translation initiation (Meyer et al., 2015). It is noteworthy that in this study, the distribution of m^6^A peaks in transcripts from tomato anthers were all enriched in stop codons and 3′-UTRs under NT and MLT conditions (Figure 4), suggesting that low temperature stress affects m6A levels of transcripts but not the m6A distribution regions on the transcripts.

ABA plays vital roles in developmental processes and mediates plant response to abiotic stress. Evidence supports that ABA signaling pathways involved in abiotic stress response differ between reproductive organs and vegetative tissues (Sharma and Nayyar, 2016). During vegetative development, increased ABA levels improve abiotic stress tolerance; exogenous ABA application enhances cold tolerance in citrus leaves, and up-regulation of ABA biosynthesis genes enhances drought resistance in petunia seedlings (Melgoza et al., 2014; Estrada-Melo et al., 2015). Previous studies revealed a negative relationship between ABA content in anthers and pollen fertility under cold stress (Sharma and Nayyar, 2016). Consistently, in our study, ABA levels were significantly increased in MLT anthers at the tetrad stage (Figure 7B). *SlPYLs* and *SlPP2Cs* gene families encode ABA signal transduction core components during tomato development and abiotic stress. ABA perception by the PYLs proteins suppressed PP2C-meidated dephosphorylation of the SnRKs and allows their activate ABA responsive genes (Raghavendra et al., 2017). Here, expression levels of ABA signal transduction core genes, *SlPYL4* was significantly downregulated and *SlPP2C1* and *SlPP2C3* were significantly upregulated by MLT stress (Supplementary Figure 6), suggesting that the ABA signal pathway regulates anther development under MLT stress. Numerous studies revealed that ABA accumulation in rice anthers under cold stress suppresses the expression of the tapetum-specific gene *OsINV4* and of sugar transport genes. It disturbs tapetum PCD, resulting in pollen abortion (Oliver et al., 2005; Ji et al., 2011). Previous studies have also reported that tomato anther carbohydrate levels were alerted responding to temperature stress (Pressman et al., 2002; Firon et al., 2006). Surprisingly, nearly all *SlCWINs* genes in our study were significantly downregulated under MLT stress. *SlCWIN7*, the rice homolog to *OsINV4*, was dramatically downregulated at both tetrad and early uninucleate stages (Figure 7C), which might distort anther sugar metabolism and, consequently, lead to pollen sterility in tomato (Figure 1).

Baron et al. (2012) reported that enhancing ABA biosynthetic gene expression or decreasing ABA transport gene expression leads to increased ABA content in *Arabidopsis* inflorescence meristems under cold stress (Baron et al., 2012). Moreover, the tomato gene, *SlABCG31*, may participate in ABA transport and is predominantly expressed in anthers (Figure 7A); however, it was significantly downregulated at the tetrad stage under MLT stress. In addition, we found an enhancement in its m^6^A methylation under MLT stress, suggesting that the decrease in *SlABCG31* transcript levels might result from its high m^6^A methylation (Figure 6). Down-regulation of *SlABCG31* results in high ABA content in tomato anthers and disturbs sugar metabolism, which may be associated with pollen abortion (Figure 8). Furthermore. in *Arabidopsis AtABCG31* also participates in the accumulation of sterol for pollen coat formation, and the *Atatabcg31* mutant displays collapsed or sticking pollen grains (Choi et al., 2014). Interestingly, in our study, tomato MLT-treated pollen grains also displayed severe collapse and aggregation phenotypes, with differences in pollen coat sculptured surfaces compared to that in NT conditions, which resembles the *Atatabcg31* mutant (Figure 1B).

In conclusion, this study reveals a unique property of anther growth under MLT stress. MLT-induced pollen abortion is the consequence of disrupted microgametogenesis, tapetum degeneration, and pollen wall formation, which may be related to differential m^6^A modification of transcripts (Figure 8). m^6^A modification is associated with ABA transport in anthers or sterol accumulation for pollen wall formation by targeting the ATP-binding cassette G gene, *SlABCG31*. Considering that pollen development and m^6^A methylation are complex processes, the molecular mechanisms behind m^6^A methylation function in pollen development under temperature stress should be further studied.

## Data Availability Statement

The datasets presented in this study can be found in online repositories. The names of the repository/repositories and accession number(s) can be found in the article/Supplementary Material.

## Author Contributions

GL and DY conceived the study. DY, HX, YL, and ML carried out the experiment. DY, XX, and MA performed the data analysis. DY and GL wrote the article. All authors discussed and commented on the final manuscript.

## Conflict of Interest

The authors declare that the research was conducted in the absence of any commercial or financial relationships that could be construed as a potential conflict of interest.

## Footnotes

1 http://homer.ucsd.edu/homer/

## Supplementary Material

Supplementary Figure 1 Schematic illustration of moderate low temperature (MLT, 10°C) stress and TUNEL assay of tapetum program cell death (PCD) in anthers. **(A)** Schematic illustration of moderate low temperature (MLT, 10°C) stress setup and sampling scheme in cultivar “Micro-Tom.” Six-week-old flowering tomato plants were exposed to MLT-stress for 6 days then moved back to normal temperature for recovery growth. The sample harvested immediately after 6 days MLT-stress were named MLT and harvested from marked tetrad anthers after recovery were named MLTR. Green and yellow lines indicate MLT-stress and NT regimes, respectively. **(B)** TUNEL assay of tapetum program cell death (PCD) in anther at tetrad, early and late uninucleate and binucleate stages under NT and MLTR conditions. The red signal is propidium iodide staining, and the green fluorescence is TUNEL positive signal. The white arrows indicate TUNEL positive signals. Scale bars = 50 μm.

Supplementary Figure 2 The expression profiles of writers, erasers and readers. **(A)** The relative levels of m^6^A in total RNA of anthers at different stages in NT condition. The levels of m^6^A in total RNA in stage-I is set to 1. I, anther at microspore mother cell stage; II, anther at tetrad stage; III, anther at early uninucleate stage; IV, anther at late uninucleate stage; V, anther at binucleate stage; VI, anther at mature pollen stage. Each value is the mean ± SD (*n* = at least 3 biological replicates with 15 plants each). **(B)** The expression of writers *in silico* data available from the tomato eFP Browser. RPKM, Reads Per Kilobase per Million mapped reads. **(C)** The expression of eraser *in silico* data available from the tomato eFP Browser. **(D)** The expression of readers *in silico* data available from the tomato eFP Browser.

Supplementary Figure 3 m^6^A-seq data analysis. **(A)** m^6^A-seq reads and mapping status with tomato genome in anther at stage-II under NT and MLT conditions. **(B)** Pearson correlation analysis of m^6^A-seq. *R*^2^ means the square of Pearson correlation. **(C)** The number of overlapped m^6^A peak-containing genes identified in three biological replicates in anthers under NT and MLT conditions, respectively. **(D)** The number of overlapped m^6^A peak-containing genes between NT and MLT anthers.

Supplementary Figure 4 KEGG analysis of different enrichment peaks related genes between NT and MLT anthers at stage-II.

Supplementary Figure 5 Comparative analysis of genes expression levels in NT and MLT conditions. **(A,B)** The expression levels of *SlPYLs* and *SlPP2Cs* genes in anther at stage-II under NT and MLT conditions. The levels of genes expression normalized to *Ubiquitin* expression are shown relative to the expression of *SlPYL2* or *SlPP2C1* in NT condition set to 1. Each value is the mean ± SD (*n* = at least three biological replicates with 15 plants each). ^∗^*P* < 0.05; ^∗∗^*P* < 0.01 (two-tailed Student’s *t-* test).

Supplementary Figure 6 Alignment of AtABCG31 and SlABCG31 full-length proteins. The multiple sequence alignment was generated using Clustal Omega. Same amino acid residues were shading with black color.

Supplementary Table 1 Primer sequences used in this study.

Supplementary Table 2 Differential m^6^A peaks and related transcripts between NT and MLT anthers.

## References

[B1] Al Mamun E. Cantrill L. C. Overall R. L. Sutton B. G. (2010). Mechanism of low-temperature-induced pollen failure in rice. *Cell Biol. Int.* 34 469–476. 10.1042/CBI20090417 20100170

[B2] Alexander M. P. (1969). Differential staining of aborted and nonaborted pollen. *Stain Technol.* 44 117–122. 10.3109/10520296909063335 4181665

[B3] Ariizumi T. Toriyama K. (2011). Genetic regulation of sporopollenin synthesis and pollen exine development. *Annu. Rev. Plant Biol.* 62 437–460. 10.1146/annurev-arplant-042809-112312 21275644

[B4] Baron K. N. Schroeder D. F. Stasolla C. (2012). Transcriptional response of abscisic acid (ABA) metabolism and transport to cold and heat stress applied at the reproductive stage of development in *Arabidopsis thaliana*. *Plant Sci.* 188–189 48–59. 10.1016/j.plantsci.2012.03.001 22525244

[B5] Barrero-Gil J. Salinas J. (2013). Post-translational regulation of cold acclimation response. *Plant Sci.* 205–206 48–54. 10.1016/j.plantsci.2013.01.008 23498862

[B6] Begcy K. Nosenko T. Zhou L. Z. Fragner L. Weckwerth W. Dresselhaus T. (2019). Male sterility in maize after transient heat stress during the tetrad stage of pollen development. *Plant Physiol.* 181 683–700. 10.1104/pp.19.00707 31378720 PMC6776839

[B7] Bodi Z. Zhong S. Mehra S. Song J. Graham N. Li H. (2012). Adenosine methylation in *Arabidopsis* mRNA is associated with the 3’- end and reduced levels cause developmental defects. *Front. Plant Sci.* 3:48. 10.3389/fpls.2012.00048 22639649 PMC3355605

[B8] Borghi L. Kang J. Ko D. Lee Y. Martinoia E. (2015). The role of ABCG-type ABC transporters in phytohormone transport. *Biochem. Soc. Trans.* 43 924–930. 10.1042/BST20150106 26517905 PMC4613532

[B9] Brukhin V. Hernould M. Gonzalez N. Chevalier C. Mouras A. (2003). Flower development schedule in tomato *Lycopersicon esculentum* cv. sweet cherry[J]. *Sex. Plant Reprod.* 15 311–320. 10.1007/s00497-003-0167-7

[B10] Chen L. Yang D. Zhang Y. Wu L. Zhang Y. Ye L. (2018). Evidence for a specific and critical role of mitogen-activated protein kinase 20 in uni-to-binucleate transition of microgametogenesis in tomato. *New Phytol.* 219 176–194. 10.1111/nph.15150 29668051

[B11] Choi H. Ohyama K. Kim Y. Y. Jin J. Y. Lee S. B. Yamaoka Y. (2014). The role of *Arabidopsis* ABCG9 and ABCG31 ATP binding cassette transporters in pollen fitness and the deposition of steryl glycosides on the pollen coat. *Plant Cell* 26 310–324. 10.1105/tpc.113.118935 24474628 PMC3963578

[B12] Criddle R. Smith B. Hansen L. (1997). A respiration-based description of plant growth rate responses to temperature. *Planta* 201 441–445. 10.1007/s004250050087

[B13] De Storme N. Geelen D. (2014). The impact of environmental stress on male reproductive development in plants: biological processes and molecular mechanisms. *Plant Cell Environ.* 37 1–18. 10.1111/pce.12142 23731015 PMC4280902

[B14] Dominissini D. Moshitch-Moshkovitz S. Salmon-Divon M. Amariglio N. Rechavi G. (2013). Transcriptome-wide mapping of N(6)-methyladenosine by m6A-seq based on immunocapturing and massively parallel sequencing. *Nat. Protocols* 8 176–189. 10.1038/nprot.2012.148 23288318

[B15] Du H. Zhao Y. He J. Zhang Y. Xi H. Liu M. (2016). YTHDF2 destabilizes m6A-containing RNA through direct recruitment of the CCR4-NOT deadenylase complex. *Nat. Commun.* 7 1–11. 10.1038/ncomms12626 27558897 PMC5007331

[B16] Duan H. C. Wei L. H. Zhang C. Wang Y. Chen L. Lu Z. (2017). ALKBH10B is an RNA N6-methyladenosine demethylase affecting *Arabidopsis* floral transition. *Plant Cell* 29 2995–3011. 10.1105/tpc.16.00912 29180595 PMC5757257

[B17] Estrada-Melo A. C. Ma C. Reid M. S. Jiang C. Z. (2015). Overexpression of an ABA biosynthesis gene using a stress-inducible promoter enhances drought resistance in petunia. *Hortic. Res.* 2 1–9. 10.1038/hortres.2015.13 26504568 PMC4595983

[B18] Fang X. Fu H. F. Gong Z. H. Chai W. G. (2016). Involvement of a universal amino acid synthesis impediment in cytoplasmic male sterility in pepper. *Sci. Rep.* 6 1–15. 10.1038/srep23357 26987793 PMC4796900

[B19] Firon N. Shaked R. Peet M. M. Pharr D. M. Zamski E. Rosenfeld K. (2006). Pollen grains of heat tolerant tomato cultivars retain higher carbohydrate concentration under heat stress conditions. *Sci. Hortic.* 109 212–217. 10.1016/j.scienta.2006.03.007

[B20] Guerra D. Crosatti C. Khoshro H. H. Mastrangelo A. M. Mica E. Mazzucotelli E. (2015). Post-transcriptional and post-translational regulations of drought and heat response in plants: a spider’s web of mechanisms. *Front. Plant Sci.* 6:57. 10.3389/fpls.2015.00057 25717333 PMC4324062

[B21] Helt G. A. Nicol J. W. Erwin E. Blossom E. Blanchard S. G. Chervitz S. A. (2009). Genoviz software development kit: java tool kit for building genomics visualization applications. *BMC Bioinformatics* 10:266. 10.1186/1471-2105-10-266 19706180 PMC2746221

[B22] Hu J. Manduzio S. Kang H. (2019). Epitranscriptomic RNA methylation in plant development and abiotic stress responses. *Front. Plant Sci.* 10:500. 10.3389/fpls.2019.00500 31110512 PMC6499213

[B23] Huong T. T. Ngoc L. N. T. Kang H. (2020). Functional characterization of a putative RNA demethylase alkbh6 in *Arabidopsis* growth and abiotic stress responses. *Int. J. Mol. Sci.* 21 1–14. 10.3390/ijms21186707 32933187 PMC7555452

[B24] Ji X. Dong B. Shiran B. Talbot M. J. Edlington J. E. Hughes T. (2011). Control of abscisic acid catabolism and abscisic acid homeostasis is important for reproductive stage stress tolerance in cereals. *Plant physiol.* 156 647–662. 10.1104/pp.111.176164 21502188 PMC3177265

[B25] Kang J. Yim S. Choi H. Kim A. Lee K. P. Lopez-Molina L. (2015). Abscisic acid transporters cooperate to control seed germination. *Nat. Commun.* 6 1–10. 10.1038/ncomms9113 26334616 PMC4569717

[B26] Kim S. Y. Hong C. B. Lee I. (2001). Heat shock stress causes stage-specific male sterility in *Arabidopsis thaliana*. *J. Plant Res.* 114 301–307. 10.1007/PL00013991

[B27] Kiran A. Kumar S. Nayyar H. Sharma K. D. (2019). Low temperature-induced aberrations in male and female reproductive organ development cause flower abortion in chickpea. *Plant Cell Environ.* 42 2075–2089. 10.1111/pce.13536 30767244

[B28] Kramer M. C. Janssen K. A. Palos K. Nelson A. D. L. Vandivier L. E. Garcia B. A. (2020). N6-methyladenosine and RNA secondary structure affect transcript stability and protein abundance during systemic salt stress in *Arabidopsis*. *Plant Direct* 4 1–22. 10.1002/pld3.239 32724893 PMC7379018

[B29] Ku S. Yoon H. Suh H. S. Chung Y. Y. (2003). Male-sterility of thermosensitive genic male-sterile rice is associated with premature programmed cell death of the tapetum. *Planta* 217 559–565. 10.1007/s00425-003-1030-7 12692728

[B30] Liu B. Mo W. J. Zhang D. De Storme N. Geelen D. (2019). Cold influences male reproductive development in plants: a hazard to fertility, but a window for evolution. *Plant Cell Physiol.* 60 7–18. 10.1093/pcp/pcy209 30602022

[B31] Liu G. Wang J. Hou X. (2020). Transcriptome-wide N6-methyladenosine (m6A) methylome profiling of heat stress in pak-choi (*Brassica rapa* ssp. chinensis). *Plants* 9 1–12. 10.3390/plants9091080 32842619 PMC7570095

[B32] Liu N. Dai Q. Zheng G. He C. Parisien M. Pan T. (2015). N(6)-methyladenosine-dependent RNA structural switches regulate RNA-protein interactions. *Nature* 518 560–564. 10.1038/nature14234 25719671 PMC4355918

[B33] Livak K. J. Schmittgen T. D. (2001). Analysis of relative gene expression data using real-time quantitative PCR and the 2-ΔΔCT method. *Methods* 25 402–408. 10.1006/meth.2001.1262 11846609

[B34] Lucca N. León G. (2012). Arabidopsis ACA7, encoding a putative auto-regulated Ca2+-ATPase, is required for normal pollen development. *Plant Cell Rep.* 31 651–659. 10.1007/s00299-011-1182-z 22044965

[B35] Luo G. Z. Macqueen A. Zheng G. Duan H. Dore L. C. Lu Z. (2014). Unique features of the m6A methylome in *Arabidopsis thaliana*. *Nat. Commun.* 5 1–8. 10.1038/ncomms6630 25430002 PMC4248235

[B36] Ma H. (2005). Molecular genetic analyses of microsporogenesis and microgametogenesis in flowering plants. *Annu. Rev. Plant Biol.* 56 393–434. 10.1146/annurev.arplant.55.031903.141717 15862102

[B37] Melgoza F. J. Kusakabe A. Nelson S. D. Melgar J. C. (2014). Exogenous applications of abscisic acid increase freeze tolerance in citrus trees. *Int. J. Fruit Sci.* 14 376–387. 10.1080/15538362.2014.899138

[B38] Meng J. Cui X. Liu H. Zhang L. Zhang S. Rao M. K. (2013). “Unveiling the dynamics in RNA epigenetic regulations,” in *IEEE International Conference on Bioinformatics and Biomedicine*, Shanghai, 139–144.

[B39] Meyer K. D. Patil D. P. Zhou J. Zinoviev A. Skabkin M. A. Elemento O. (2015). 5’ UTR m6A promotes cap-independent translation. *Cell* 163 999–1010. 10.1016/j.cell.2015.10.012 26593424 PMC4695625

[B40] Min L. Li Y. Hu Q. Zhu L. Gao W. Wu Y. (2014). Sugar and auxin signaling pathways respond to high-temperature stress during anther development as revealed by transcript profiling analysis in cotton. *Plant Physiol.* 164 1293–1308. 10.1104/pp.113.232314 24481135 PMC3938621

[B41] Müller F. Rieu I. (2016). Acclimation to high temperature during pollen development. *Plant Reprod.* 29 107–118. 10.1007/s00497-016-0282-x 27067439 PMC4909792

[B42] Oda S. Kaneko F. Yano K. Fujioka T. Masuko H. Park J. I. (2010). Morphological and gene expression analysis under cool temperature conditions in rice anther development. *Genes Genet. Syst.* 85 107–120. 10.1266/ggs.85.107 20558897

[B43] Oliver S. N. Dennis E. S. Dolferus R. (2007). ABA regulates apoplastic sugar transport and is a potential signal for cold-induced pollen sterility in rice. *Plant Cell Physiol.* 48 1319–1330. 10.1093/pcp/pcm100 17693452

[B44] Oliver S. N. Van Dongen J. T. Alfred S. C. Mamun E. A. Zhao X. Saini H. S. (2005). Cold-induced repression of the rice anther-specific cell wall invertase gene OSINV4 is correlated with sucrose accumulation and pollen sterility. *Plant Cell Environ.* 28 1534–1551. 10.1111/j.1365-3040.2005.01390.x

[B45] Omidi M. Siahpoosh M. R. Mamghani R. Modarresi M. (2014). The influence of terminal heat stress on meiosis abnormalities in pollen mother cells of wheat. *Cytologia* 79 49–58. 10.1508/cytologia.79.49

[B46] Pacini E. Dolferus R. (2019). Pollen developmental arrest: maintaining pollen fertility in a world with a changing climate. *Front. Plant Sci.* 10:679. 10.3389/fpls.2019.00679 31178886 PMC6544056

[B47] Paupière M. J. Müller F. Li H. Rieu I. Tikunov Y. M. Visser R. G. F. (2017). Untargeted metabolomic analysis of tomato pollen development and heat stress response. *Plant Reprod.* 30 81–94. 10.1007/s00497-017-0301-6 28508929 PMC5486769

[B48] Peng Z. Cheng L. He Y. J. Wang J. Guan X. Liu S. (2013). Cytological study on microsporogenesis of Solanum lycopersicum var. micro-tom under high temperature stress. *Acta Ecol. Sin.* 33 2084–2092. 10.5846/stxb201112261972

[B49] Pressman E. Peet M. M. Pharr D. M. (2002). The effect of heat stress on tomato pollen characteristics is associated with changes in carbohydrate concentration in the developing anthers. *Ann. Bot.* 90 631–636. 10.1093/aob/mcf240 12466104 PMC4240456

[B50] Raghavendra A. S. Gonugunta V. K. Christmann A. Grill E. (2017). ABA perception and signalling. *Trends Plant Sci.* 15 395–401. 10.1016/j.tplants.2010.04.006 20493758

[B51] Roundtree I. A. Luo G. Z. Zhang Z. Wang X. Zhou T. Cui Y. (2017). YTHDC1 mediates nuclear export of N6-methyladenosine methylated mRNAs. *eLife* 6 1–28. 10.7554/eLife.31311 28984244 PMC5648532

[B52] Růžička K. Zhang M. Campilho A. Bodi Z. Kashif M. Saleh M. (2017). Identification of factors required for m6A mRNA methylation in *Arabidopsis* reveals a role for the conserved E3 ubiquitin ligase HAKAI. *New Phytol.* 215 157–172. 10.1111/nph.14586 28503769 PMC5488176

[B53] Sharma K. D. Nayyar H. (2016). Regulatory networks in pollen development under cold stress. *Front. Plant Sci.* 7:402. 10.3389/fpls.2016.00402 27066044 PMC4814731

[B54] Shen L. Liang Z. Gu X. Chen Y. Teo Z. W. Hou X. (2016). N(6)-Methyladenosine RNA modification regulates shoot stem cell fate in *Arabidopsis*. *Dev. Cell* 38 186–200. 10.1016/j.devcel.2016.06.008 27396363 PMC6364302

[B55] Shi J. Cui M. Yang L. Kim Y. J. Zhang D. (2015). Genetic and biochemical mechanisms of pollen wall development. *Trends Plant Sci.* 20 741–753. 10.1016/j.tplants.2015.07.010 26442683

[B56] Sun L. Sui X. Lucas W. J. Li Y. Feng S. Ma S. (2019). Down-regulation of the sucrose transporter cssut1 causes male sterility by altering carbohydrate supply. *Plant Physiol.* 180 986–997. 10.1104/pp.19.00317 30967482 PMC6548282

[B57] Tomato Genome Consortium (2012). The tomato genome sequence provides insights into fleshy fruit evolution. *Nature* 485 635–641. 10.1038/nature11119 22660326 PMC3378239

[B58] Vespa L. Vachon G. Berger F. Perazza D. Faure J. D. Herzog M. (2004). The immunophilin-interacting protein AtFIP37 from *Arabidopsis* is essential for plant development and is involved in trichome endoreduplication. *Plant Physiol.* 134 1283–1292. 10.1104/pp.103.028050 15047892 PMC419804

[B59] Wang M. Hoekstra S. van Bergen S. Lamers G. E. Oppedijk B. J. van der Heijden M. W. (1999). Apoptosis in developing anthers and the role of ABA in this process during androgenesis in *Hordeum vulgare* L. *Plant Mol. Biol.* 39 489–501. 10.1023/a:1006198431596 10092177

[B60] Wang X. Lu Z. Gomez A. Hon G. C. Yue Y. N. Han D. L. (2014). N6-methyladenosine-dependent regulation of messenger RNA stability. *Nature* 505 117–120. 10.1038/nature12730 24284625 PMC3877715

[B61] Xiao W. Adhikari S. Dahal U. Chen Y. S. Hao Y. J. Sun B. F. (2016). Nuclear m6A reader YTHDC1 regulates mRNA splicing. *Mol. Cell* 61 507–519. 10.1016/j.molcel.2016.01.012 26876937

[B62] Yang S. Sweetman J. P. Amirsadeghi S. Barghchi M. Huttly A. K. Chung W. I. (2001). Novel anther-specific myb genes from tobacco as putative regulators of phenylalanine ammonia-lyase expression. *Plant Physiol.* 126 1738–1753. 10.1104/pp.126.4.1738 11500571 PMC117172

[B63] Yue H. Nie X. Yan Z. Weining S. (2019). N6-methyladenosine regulatory machinery in plants: composition, function and evolution. *Plant Biotechnol. J.* 17 1194–1208. 10.1111/pbi.13149 31070865 PMC6576107

[B64] Yue Y. Liu J. He C. (2015). RNA N6-methyladenosine methylation in post-transcriptional gene expression regulation. *Genes Dev.* 29 1343–1355. 10.1101/gad.262766.115 26159994 PMC4511210

[B65] Zhang F. Zhang Y. C. Liao J. Y. Yu Y. Zhou Y. F. Feng Y. Z. (2019). The subunit of RNA n6-methyladenosine methyltransferase OsFIP regulates early degeneration of microspores in rice. *PLoS Genet.* 15:e1008120. 10.1371/journal.pgen.1008120 31116744 PMC6548400

[B66] Zhang Y. Liu T. Meyer C. A. Eeckhoute J. Johnson D. S. Bernstein B. E. (2008). Model-based analysis of ChIP-Seq (MACS). *Genome Biology* 9 R137. 10.1186/gb-2008-9-9-r137 18798982 PMC2592715

[B67] Zhao B. S. Roundtree I. A. He C. (2017). Post-transcriptional gene regulation by mRNA modifications. *Nat. Rev. Mol. Cell Biol.* 18 31–42. 10.1038/nrm.2016.132 27808276 PMC5167638

[B68] Zhao G. Shi J. Liang W. Zhang D. (2016). ATP binding cassette G transporters and plant male reproduction. *Plant Signal. Behav.* 11 1–6. 10.1080/15592324.2015.1136764 26906115 PMC4883977

[B69] Zhong S. Li H. Bodi Z. Button J. Vespa L. Herzog M. (2008). MTA is an *Arabidopsis* messenger RNA adenosine methylase and interacts with a homolog of a sex-specific splicing factor. *Plant Cell* 20 1278–1288. 10.1105/tpc.108.058883 18505803 PMC2438467

[B70] Zhou J. Wan J. Gao X. Zhang X. Jaffrey S. R. Qian S. B. (2015). Dynamic m6A mRNA methylation directs translational control of heat shock response. *Nature* 526 591–594. 10.1038/nature15377 26458103 PMC4851248

[B71] Zhou L. Tian S. Qin G. (2019). RNA methylomes reveal the m6A-mediated regulation of DNA demethylase gene SlDML2 in tomato fruit ripening. *Genome Biol.* 20 1–23. 10.1186/s13059-019-1771-7 31387610 PMC6683476

[B72] Zhu J. K. (2016). Abiotic stress signaling and responses in plants. *Cell* 167 313–324. 10.1016/j.cell.2016.08.029 27716505 PMC5104190

