# Supplementary figures and images for: RNA N6-Methyladenosine Responds to Low-Temperature Stress in Tomato Anthers
Source: Front Plant Sci. 2021 Jun 4;12:687826. doi: 10.3389/fpls.2021.687826 (PMC8213351; doi:10.3389/fpls.2021.687826)

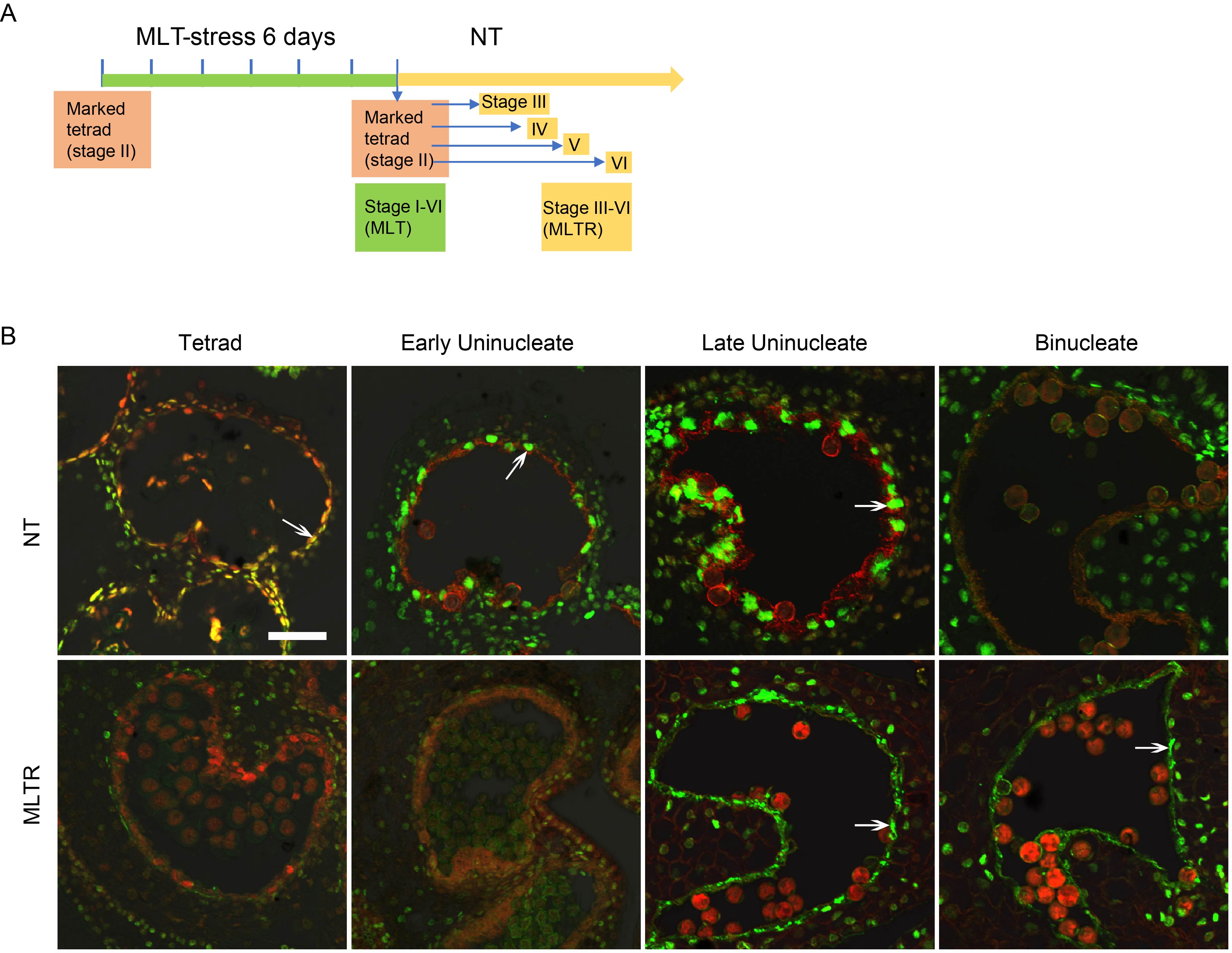

Supplement: Supplementary Figure 1 — Schematic illustration of moderate low temperature (MLT, 10°C) stress and TUNEL assay of tapetum program cell death (PCD) in anthers. (A) Schematic illustration of moderate low temperature (MLT, 10°C) stress setup and sampling scheme in cultivar “Micro-Tom.” Six-week-old flowering tomato plants were exposed to MLT-stress for 6 days then moved back to normal temperature for recovery growth. The sample harvested immediately after 6 days MLT-stress were named MLT and harvested from marked tetrad anthers after recovery were named MLTR. Green and yellow lines indicate MLT-stress and NT regimes, respectively. (B) TUNEL assay of tapetum program cell death (PCD) in anther at tetrad, early and late uninucleate and binucleate stages under NT and MLTR conditions. The red signal is propidium iodide staining, and the green fluorescence is TUNEL positive signal. The white arrows indicate TUNEL positive signals. Scale bars = 50 μm. [file Image_1.jpg]

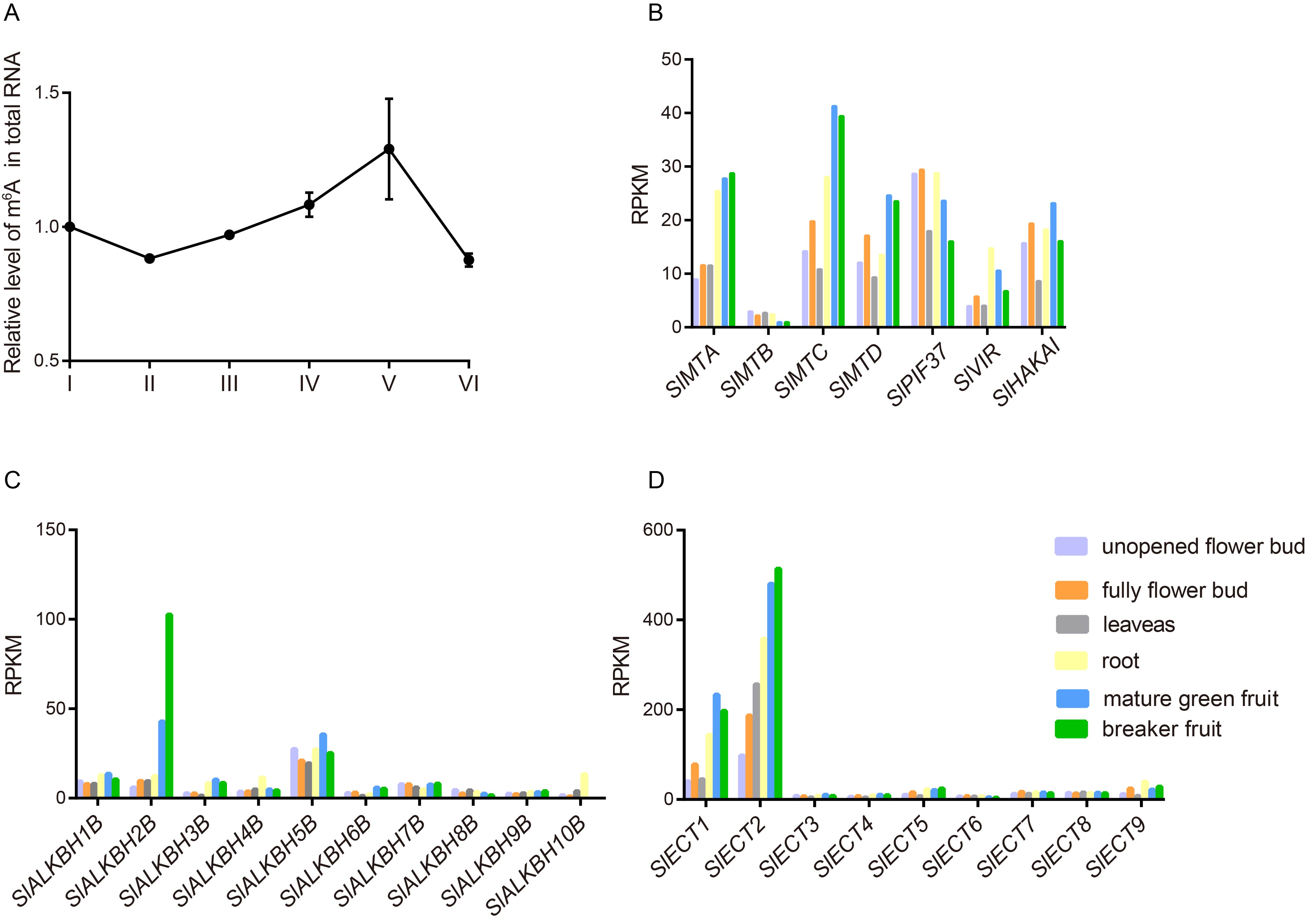

Supplement: Supplementary Figure 2 — The expression profiles of writers, erasers and readers. (A) The relative levels of m6A in total RNA of anthers at different stages in NT condition. The levels of m6A in total RNA in stage-I is set to 1. I, anther at microspore mother cell stage; II, anther at tetrad stage; III, anther at early uninucleate stage; IV, anther at late uninucleate stage; V, anther at binucleate stage; VI, anther at mature pollen stage. Each value is the mean ± SD (n = at least 3 biological replicates with 15 plants each). (B) The expression of writers in silico data available from the tomato eFP Browser. RPKM, Reads Per Kilobase per Million mapped reads. (C) The expression of eraser in silico data available from the tomato eFP Browser. (D) The expression of readers in silico data available from the tomato eFP Browser. [file Image_2.JPEG]

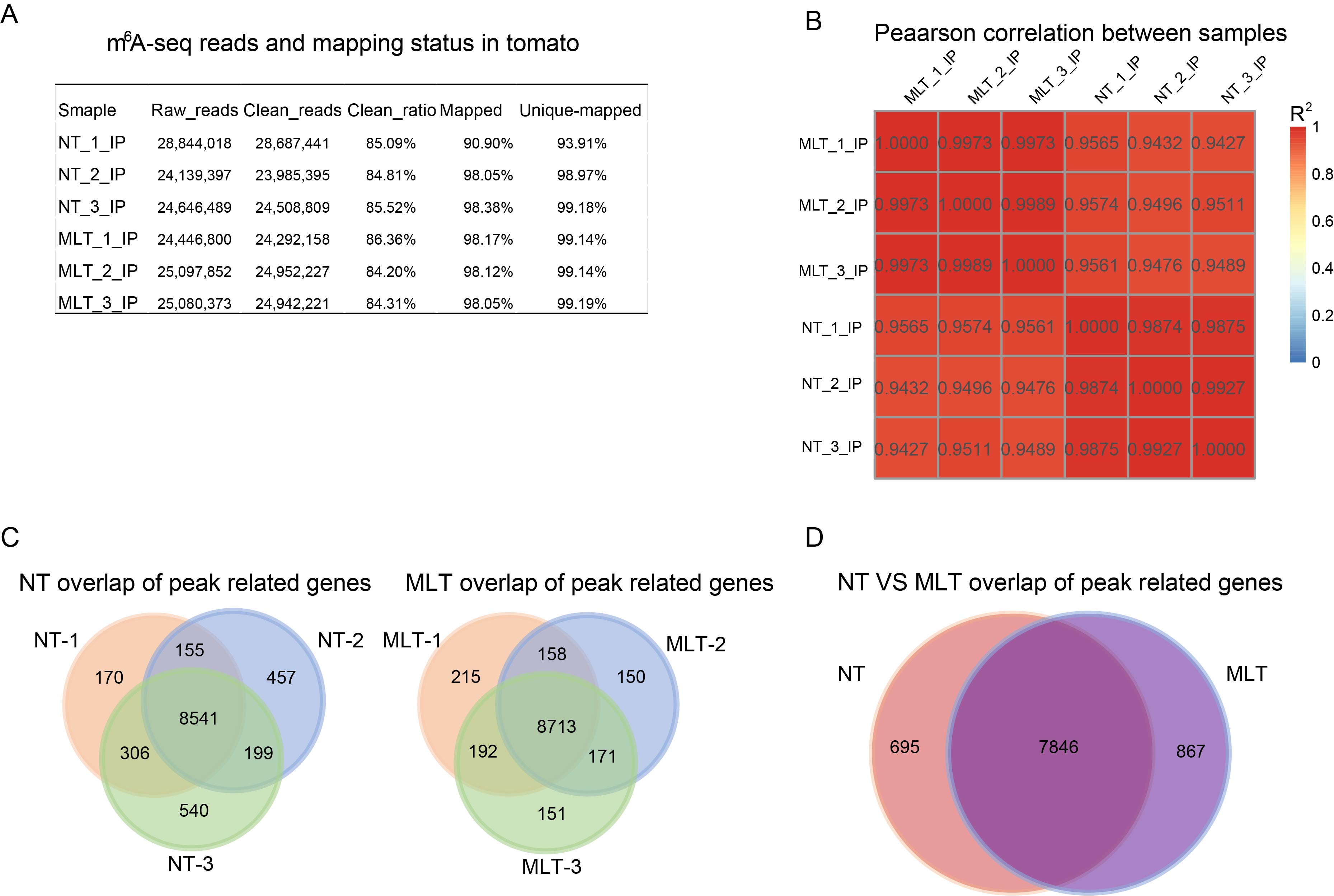

Supplement: Supplementary Figure 3 — m6A-seq data analysis. (A) m6A-seq reads and mapping status with tomato genome in anther at stage-II under NT and MLT conditions. (B) Pearson correlation analysis of m6A-seq. R2 means the square of Pearson correlation. (C) The number of overlapped m6A peak-containing genes identified in three biological replicates in anthers under NT and MLT conditions, respectively. (D) The number of overlapped m6A peak-containing genes between NT and MLT anthers. [file Image_3.JPEG]

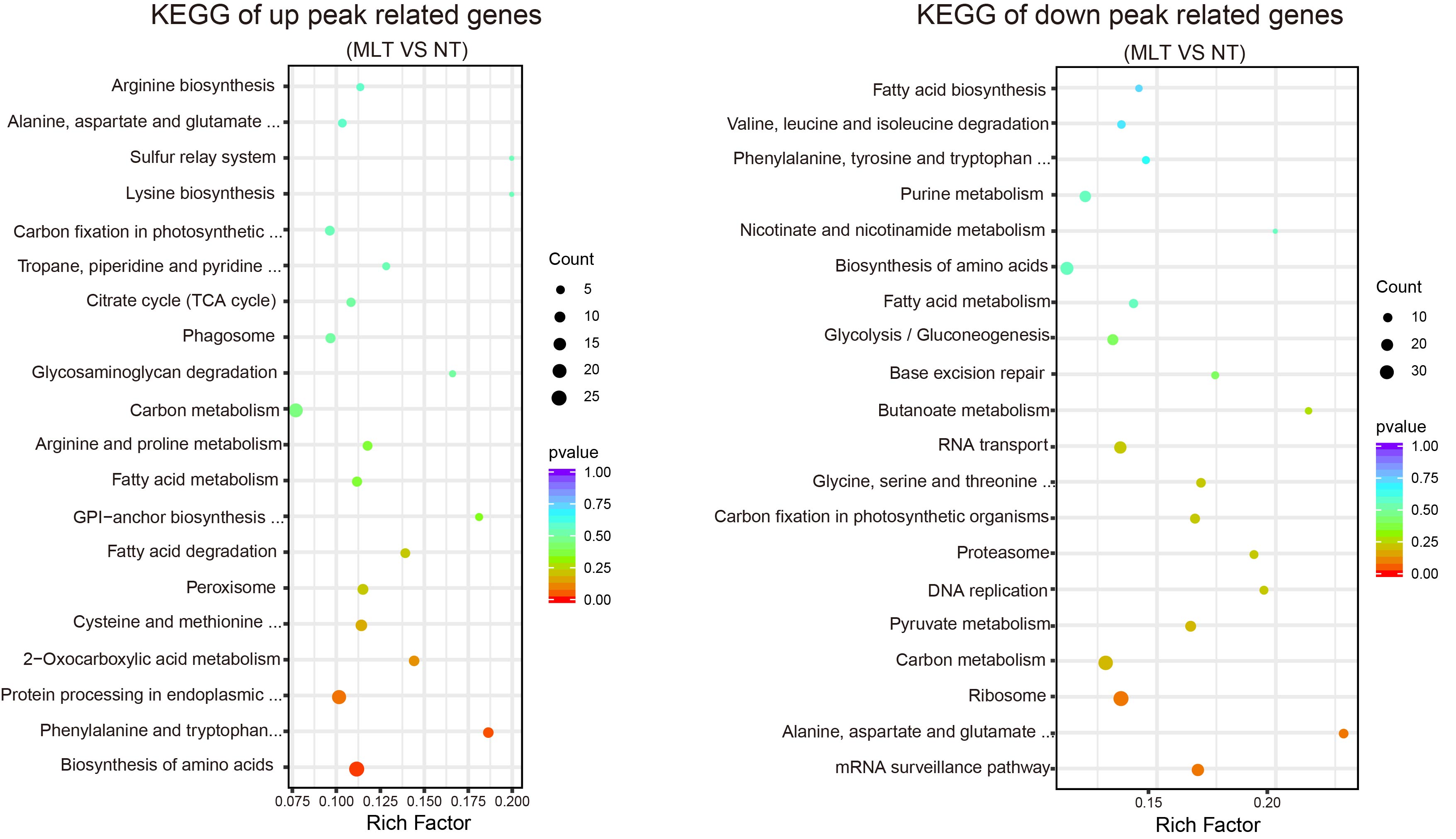

Supplement: Supplementary Figure 4 — KEGG analysis of different enrichment peaks related genes between NT and MLT anthers at stage-II. [file Image_4.JPEG]

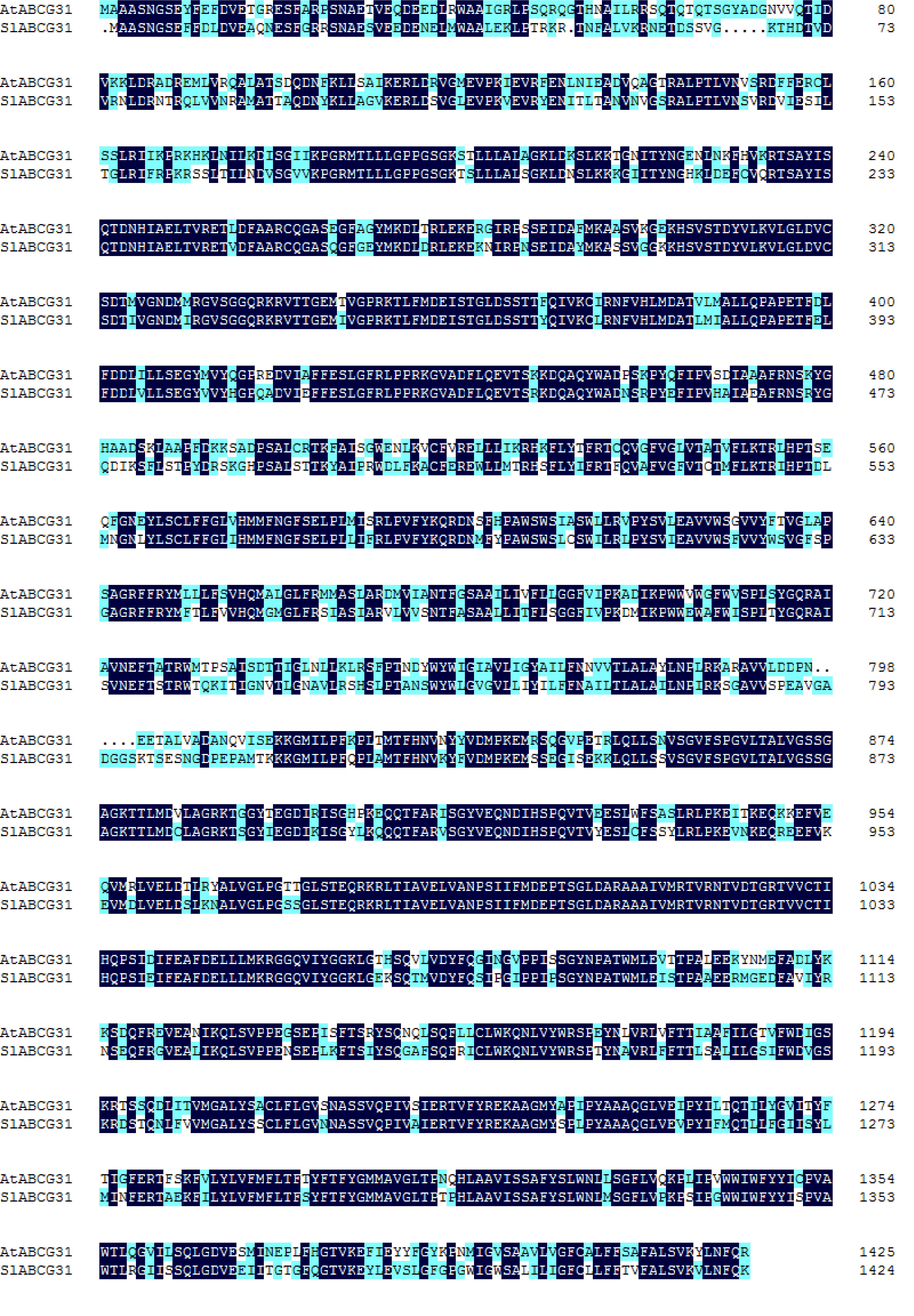

Supplement: Supplementary Figure 5 — Comparative analysis of genes expression levels in NT and MLT conditions. (A,B) The expression levels of SlPYLs and SlPP2Cs genes in anther at stage-II under NT and MLT conditions. The levels of genes expression normalized to Ubiquitin expression are shown relative to the expression of SlPYL2 or SlPP2C1 in NT condition set to 1. Each value is the mean ± SD (n = at least three biological replicates with 15 plants each). ∗P < 0.05; ∗∗P < 0.01 (two-tailed Student’s t- test). [file Image_5.JPEG]

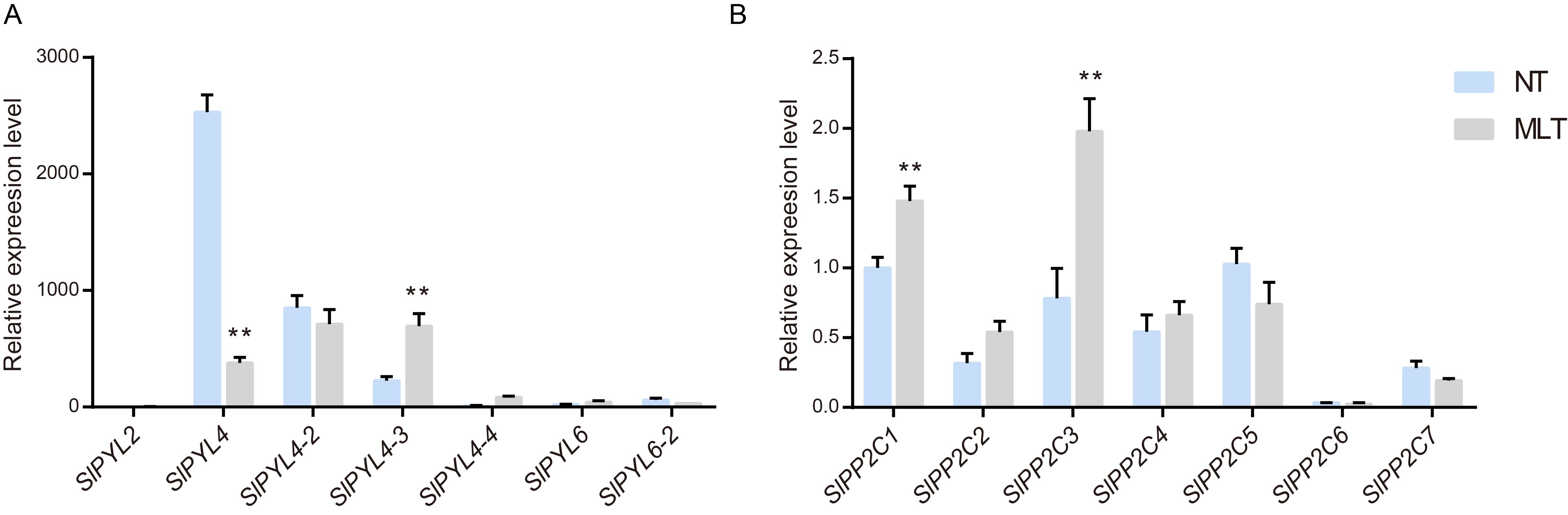

Supplement: Supplementary Figure 6 — Alignment of AtABCG31 and SlABCG31 full-length proteins. The multiple sequence alignment was generated using Clustal Omega. Same amino acid residues were shading with black color. [file Image_6.JPEG]
